# Ligand-Promoted Surface Solubilization of TiO_2_ Nanoparticles by the Enterobactin Siderophore in Biological Medium

**DOI:** 10.3390/biom12101516

**Published:** 2022-10-19

**Authors:** Jérôme Laisney, Mireille Chevallet, Caroline Fauquant, Camille Sageot, Yohann Moreau, Daniela Predoi, Nathalie Herlin-Boime, Colette Lebrun, Isabelle Michaud-Soret

**Affiliations:** 1 Université Grenoble Alpes, CNRS CEA, IRIG-LCBM, 38000 Grenoble, France; 2 National Institute of Materials Physics, Atomistilor 105 bis, 077125 Magurele, Romania; 3 Université Paris-Saclay, NIMBE, CEA, CNRS, CEA Saclay, 91191 Gif-sur-Yvette, France; 4 Université Grenoble Alpes, IRIG-SyMMES, CEA, CNRS, CEA-Grenoble, 38000 Grenoble, France

**Keywords:** titanium dioxide, nanoparticle, siderophore, enterobactin, ligand-promoted dissolution, biological medium, E171, *Escherichia coli*

## Abstract

Titanium dioxide nanoparticles (TiO_2_-NPs) are increasingly used in consumer products for their particular properties. Even though TiO_2_ is considered chemically stable and insoluble, studying their behavior in biological environments is of great importance to figure their potential dissolution and transformation. The interaction between TiO_2_-NPs with different sizes and crystallographic forms (anatase and rutile) and the strong chelating enterobactin (**ent**) siderophore was investigated to look at a possible dissolution. For the first time, direct evidence of anatase TiO_2_-NP surface dissolution or solubilization (i.e., the removal of Ti atoms located at the surface) in a biological medium by this siderophore was shown and the progressive formation of a hexacoordinated titanium–enterobactin (Ti–**ent**) complex observed. This complex was characterized by UV–visible and Fourier transform infrared (FTIR) spectroscopy (both supported by Density Functional Theory calculations) as well as electrospray ionization mass spectrometry (ESI-MS) and X-ray photoelectron spectroscopy (XPS). A maximum of ca. 6.3% of Ti surface atoms were found to be solubilized after 24 h of incubation, releasing Ti–**ent** complexes in the micromolar range that could then be taken up by bacteria in an iron-depleted medium. From a health and environmental point of view, the effects associated to the solubilization of the E171 TiO_2_ food additive in the presence of enterobactin and the entrance of the Ti–enterobactin complex in bacteria were questioned.

## 1. Introduction

**Figure 1:**
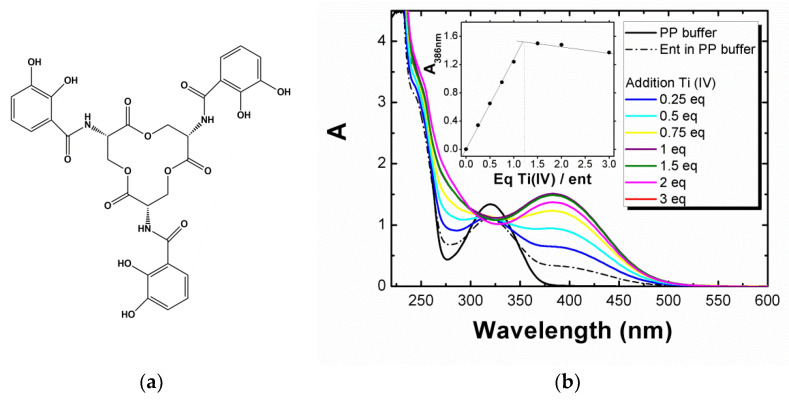
(**a**) Molecular structure of the enterobactin siderophore (**ent**) and (**b**) enterobactin metallation (100 µM) followed by UV–visible spectroscopy by successive additions of Ti(IV) equivalent (TiCl_4_, 1 mM) in the PP medium. (Inserts) Determination of the stoichiometry of the Ti–enterobactin complex by following the evolution of the absorbance at 386 nm during addition of Ti(IV). A = absorbance.

Among its many possible applications, titanium dioxide (TiO_2_) is commonly used in the industry as a white pigment for its brightness, opacifying power, and very high refractive index, but also for its interesting photocatalytic properties [1,2]. With the emergence of nanotechnology and its new field of possibilities, the nanoparticulate forms of TiO_2_ (TiO_2_-NPs) are found in an increasing number of daily-life products [3,4]**,** such as adhesives, paints, sunscreens, toothpastes, and cosmetics, as well as in the food industry [5,6]. Consequently, their production has reached more than 10,000 tons worldwide [7,8] and it is expected to grow exponentially in order to reach several millions of tons in 2025 [9]. For example, the TiO_2_ food additive E171 used to make foods whiter and brighter contains a variable portion of TiO_2_-NPs, depending on the source (36% in Weir et al.) [10]. The average ingested amount of titanium has been estimated in the UK at about 5.4 mg per person per day [11,12]. A more recent study gives 0.2 to 1 mg/kg body weight/day for adults and 1 to 3 mg/kg body weight/day for children [10]. TiO_2_ is considered robust, chemically stable, and, by common belief, insoluble. Thus, very few studies [13] have focused on the dissolution of TiO_2_ (none conducted in a biological medium to the best of our knowledge) that could potentially have an impact on its antimicrobial properties, toxicity, medicinal applications, and environmental fate [14,15]. Whereas size is considered as the primary physicochemical property affecting the solubility of NPs, various other parameters, such as surface area, morphology, crystallinity and crystal structure, and presence of impurities or ad-atoms, must also be considered [14]. The presence of organic ligands can also affect suspension stability and lead to the dissolution of the NPs [16,17]. Among them, siderophores are low molecular weight organic ligands secreted by bacteria to capture iron essential for their development [18]. They possess an extremely high affinity for iron (III) [19]**,** with association constants (K_a_) ranging from 10^30^ to 10^52^ M**^−^**^1^, and display high structural diversity [20,21]. Most of them bind Fe^3+^ ions via hydroxamate, catecholate, or hydroxycarboxylate groups [20,22,23]. In particular, enterobactin (**ent**) forms one of the most stable complexes ever reported with iron [20] (K_f_ = 10^49^) and is involved in iron transport in Gram-negative bacteria such as *Escherichia coli*. Enterobactin’s structure (Figure 1a) is composed of three catechol groups linked to a central lactone macrocycle. The affinity with Fe(III) is so high that enterobactin is able to solubilize iron present in minerals such as olivine [22,24]. At a low pH, enterobactin coordinates iron in a “salicylate mode” via an ortho carbonyl group and only one phenolic oxygen, the other one being protonated [25,26]. However, in the neutral and alkaline pH range, catecholate groups of enterobactin form five membered rings with iron via the phenolic oxygens after loss of two protons to bind metals in a so-called “catecholate mode”. In the ionic form, Ti(IV) and Fe(III) have an almost identical ionic radius (68 pm and 64 pm, respectively) [27]. Moreover, Ti(IV) is a stronger hard Lewis acid than Fe(III) (the log K1 for OH- binding is 14.33 [28] compared to 11.21 for Fe(III)) [29] and favors octahedral environments [30,31]. Ti(IV), therefore, has a particular affinity for oxygenated ligands, and could compete for hexacoordinating oxygenated metalloproteins or biomolecules [27]. Thus, we expected enterobactin to bind covalently and form complexes with Ti(IV), as seen with transferrin [32] or TiO_2_ NP surface modified with dopamine derivatives [33]. Few studies have reported the high affinity of Ti(IV) for triscatecholate groups (K_a_ = 10^60^), but the binding of enterobactin to TiO_2_ films has already been demonstrated [34,35,36]. More recently, another study from Baramov et al. [37] reported the possibility for enterobactin to bind titanium and other metals such as silicium and germanium, to form hexacoordinated complexes. However, the binding of enterobactin on TiO_2_-NPs and their possible dissolution have not yet been described.

In the present work, we demonstrate that enterobactin is able to interact with TiO_2_-NPs, binding to their oxide surface, but also promote Ti(IV) solubilization via the formation of Ti–**ent** complexes. Here, we present how this ligand-promoted surface solubilization of titanium from TiO_2_-NPs is influenced by intrinsic properties such as the size, surface defects, and crystallographic form of the particles. We questioned the dissolution of the E171 TiO_2_ food additive and the entrance of the Ti–**ent** complex into *Escherichia coli* bacteria, raising questions about possible environmental and heath impact of the interaction of a strong iron chelator such as enterobactin secreted by bacteria and TiO_2_-NPs.

## 2. Materials and Methods

### 2.1. Chemicals and Instruments

**Chemicals and TiO_2_ nanoparticles**. Enterobactin (purity > 98%) and all the chemicals were purchased from Sigma (Burlington, MA, USA) and used without further purification. The homemade nanoparticles were synthesized by laser pyrolysis according to the procedure previously described [38,39,40]. Titanium tetraisopropoxide (97% purity) was the precursor for TiO_2_ synthesis. All commercially available and homemade TiO_2_ nanoparticles (TiO_2_-NPs) used in this study are listed in Table S1 (Supplementary Materials).

**UV-Vis spectroscopy.** The absorption spectra were recorded at room temperature (RT) with a quartz cuvette (100 or 500 μL; 1 cm optical path) on a Shimazu spectrophotometer (Kyoto, Japan).

**Dynamic Light Scattering.** Average diameter, % intensity, % number, and polydispersity of the nanoparticle suspensions were determined by dynamic light scattering (DLS) using a DynaPro NanoStar from Wyatt Technology (Santa Barbara, CA, USA). The suspensions of NPs were diluted in ultrapure water at 10 μg/mL prior to measurement.

**Mass Spectrometry.** ESI-MS mass spectra were carried out at RT using a LXQ type mass spectrometer from Thermo Scientific (Waltham, MA, USA) equipped with an electrospray ionization source (ESI) and an ion trap-type analyzer (linear trap). The solutions were injected into the spectrometer using a syringe pump at a flow rate of 10 μL/min through silica tubing. The ionization voltage was of the order of 2 kV and the temperature of the transfer capillary was set at 250 °C.

**ICP-AES.** The amount of solubilized titanium was accurately measured by inductively coupled plasma atomic emission spectroscopy (ICP-AES) using an ICPE-9000 instrument from Shimadzu Scientific Instruments. The protocol consisted of the dilution of 250 µL of supernatants in 460 µL of pure nitric acid (65%). An Ytterbium internal standard was added, and the samples were further diluted in pure water, qs 6.0 mL, prior to analysis. Calibration was done from a standard curve of an atomic absorption standard solution of titanium (Sigma-Aldrich, St. Louis, MO, USA).

**FTIR spectroscopy.** The FTIR absorption spectra were performed at RT on a Spectrum 100 Perkin Elmer spectrometer (PerkinElmer, Waltham, MA, USA). The data acquisition was performed both in solution with the supernatant using a CaF_2_ cell adapted for liquid and in the solid state (KBr pellet), with the dried powders collected by ultracentrifugation. The 2 cm^−1^-resolution spectra were obtained after 200 scans in the 1700–950 cm^−1^ range in solution because of the water absorption or in the 4000–500 cm^−1^ range (KBr pellets). 

**Theoretical calculations.** Details of the optimization of the structures and calculation of the simulated UV–visible and infrared spectra can be found in Supplementary Materials, Section A.

**X-ray photoelectron spectroscopy (XPS) analysis.** The interaction of **ent** with the TiO_2_-NPs surface was determined by XPS (Supplementary Materials, Section B) using a 1486.61 eV Al Kα monochromatic X-ray source (SPECS GmbH, Berlin, Germany). Survey (0–1400 cm^−1^) and high-resolution (C1s, Ti2p, O1s, N1s and P2p) spectra were collected from the dried nanocomposite powders. The elemental composition (atomic percentage of each element) was calculated from the high-resolution spectra using Casa XPS 2.3.14 software. The calibration of the binding energy was carried out from the C1s peak at 285 eV (C-C/C-H hydrocarbon bonds).

### 2.2. Protocols

**Metallation of enterobactin by titanium salts.** Metallation of enterobactin was carried out in water (pH 7.0) and in pyrophosphate neutral buffered PP medium (glucose (0.4 wt%), MgSO_4_ (50 µM), casamino acids (0.1%), and Na_4_P_2_O_7_ (5 mM); pH 7.5) by successive addition of equivalents of titanium (IV) (TiCl_4_, 1 mM) followed by absorption at 386 nm (ε_386nm_ = 11,080 M ^−1^.cm^−1^).

**Preparation of nanoparticles suspensions prior to incubation.** In a round-bottom centrifuge tube, 20 mg of the TiO_2_ nanoparticles were weighed and put in suspension in 2 mL of ultrapure water ([TiO_2_] = 10 mg/mL). The suspension was sonicated for 1 h at 5 °C (pulse of 100 W, 1s on/1s off, average energy deposited: 150 kJ) in a cup-horn system to avoid contact with the probe and reduce aerosolization (Bioblock Scientific, Illkirch, France; Vibra cell 75041).

**Incubation of TiO_2_-NP by enterobactin.** The TiO_2_-NP suspension (10 mg/mL in 2 mL of ultrapure water) was diluted in PP medium ([TiO_2_-NP] = 5mg/mL, 4mL). A total of 2 mL of the suspension was used as a control to measure the amount of free titanium present in the suspension ([Ti]_0_) while, in the remaining 2 mL, enterobactin was introduced ([**ent**]_f_ = 50 μM). Both samples were then incubated in the dark at 37 °C under stirring (200 rpm) for 24 h. After incubation, the suspensions were ultracentrifuged at 75,000 rpm during 45 min at 5 °C. The supernatants were then filtered on a 3 kDa Centricon unit with cellulose membranes to remove all the eventual remaining nanoparticles while the pellets were washed with water to get rid of unreacted enterobactin species from the surface. The powder was finally dried at 50 °C overnight in an oven before characterization.

**Influence of concentration on the solubilization rate of TiO_2_-NP.** This set of experiments was conducted with the same preparation of TiO_2_-A12 NPs. For this, 50 mg of TiO_2_-A12 NPs were weighed and introduced in a round-bottom centrifuge tube for sonication 1 h at 5 °C with 5 mL ultrapure water ([TiO_2_-A12 NP] = 10 mg/mL). The suspension was diluted in PP buffer ([TiO_2_-A12 NP] = 5 mg/mL, 10 mL) and then divided into 5 distinct experiments, where enterobactin was added to reach final concentrations from 0 to 100 µM. After incubation for 24 h at 37 °C, the suspensions were treated as previously described.

**Influence of the incubation time on the solubilization of TiO_2_-NP.** Similarly, 60 mg of TiO_2_-A12 NP were dispersed in 6 mL ultrapure water ([TiO_2_-A12] = 10 mg/mL) and then sonicated for 1 hour at 5 °C following the same procedure. The suspension was diluted in PP medium ([TiO_2_-A12] = 5 mg/mL, 12 mL) and separated into 6 different experiments in which enterobactin was introduced ([**ent**]_f_ = 50 µM). These experiments were then incubated at 37 °C, from 0 to 144 h. After incubation, the suspensions were treated as previously described.

**Determination of the enterobactin concentration in the supernatant by the Arnow method.** Concentration of enterobactin present in the supernatant was quantified by UV–visible spectroscopy by the determination of its catechol groups according to the Arnow method [41,42]. In total, 100 μL of supernatant were incubated under stirring in the presence of HCl (0.5 M), 100 μL of the Arnow reagent (10 g of NaNO_2_ and 10 g of sodium molybdate solubilized in 100 mL of ultrapure water), and 100 μL of NaOH (1 M). The pink-colored solutions obtained were then transferred to black plates. The OD measurement (between 400 and 700 nm) was measured instantaneously by UV–visible spectroscopy (TECAN).

**Dosage of titanium in bacteria in the presence or absence of enterobactin, in complete or iron-depleted medium.** Precultures of bacteria (BL21 strain) were performed overnight at 37 °C, under agitation, in Luria-Bertini (LB) or PP medium. PP and LB medium were inoculated at 0.1 OD 600, and 0 or 3.5 µM of the preformed Ti–**ent** complexes was added to each culture and incubated at 37 °C under agitation. Cultures were stopped after 3 to 4 h before reaching the plateau. OD 600 was measured to quantify the bacteria density. Bacteria were rinsed in a 50 mM pH 7.5 Tris buffer and the bacteria pellets were suspended with 100 µL of pure nitric acid and mineralized overnight at 95 °C in DigiPreps (SCP Science). The internal standard Ytterbium was added, and the samples were diluted in pure water, qs 6.0 mL, prior to analysis by ICP-AES. The volume of one bacterium was set at 2 femtoliters to calculate concentrations. The experiments were done 4 times and the results are given as averages over the 4 experiments.

## 3. Results

### 3.1. Complexation of Enterobactin with Ti(IV) Salts

The spectroscopic signature of the complex formed by the metallation of enterobactin by titanium (IV) salts in a biological medium was recorded by UV–visible spectroscopy by adding increasing amounts of Ti(IV) equivalents to a solution containing enterobactin (100 μM) in pyrophosphate neutral buffer medium (PP) (see experimental conditions in Materials and Methods). After addition, a yellow complex was formed instantaneously in the PP medium (Figure 1b) and in water (Figure S1) at ambient temperature, regardless the amount of titanium added. This yellow complex possessed a defined spectroscopic signature consisting of a transition band at 386 nm (ε_386nm_ = 11,080 L.mol^−1^.cm^−1^). Theoretical UV–visible spectra (see computational details and Figure S9, Supplementary Materials, Section A) were computed by the mean of the TD-DFT methodology for the metal-free enterobactin and salicylate/catecholate coordinating mode of Ti(IV), with enterobactin in a protonation state corresponding to pH = 7. Compared to the metal-free enterobactin, both the salicylate and catecholate forms show an additional transition around 400 nm, which can be related to the one observed experimentally at 386 nm upon metallation of enterobactin. For both forms, this band was attributed to an oxygen to Ti LMCT (ligand-to-metal charge transfer) transition. Hence, we considered this band as characteristic of the formation of a titanium–enterobactin complex. However, the very close values and the precision of our calculations did not allow us to unambiguously identify which of the salicylate or catecholate forms were predominant in solution at this pH range. Furthermore, all our calculations yielded a transition at around 300 to 325 nm for the metalated and metal-free enterobactin, consistent with an LLCT transition (ligand-to-ligand charge transfer), characteristic of the enterobactin ligand. The stoichiometry of the Ti–**ent** complex was determined by titration of enterobactin as a function of the concentration of TiCl_4_ added by following the evolution of the absorption band at 386 nm. It emerged that one molecule of enterobactin is able to bind around 1 equivalent of titanium (IV) (see Insert, Figure 1b). Electrospray ionization mass spectrometry (ESI-MS) confirmed the metallation of enterobactin (**ent**H_6_) by titanium (Figure S2 in Supplementary Materials). The experimental spectrum in negative mode was in agreement with the theoretical spectrum and contained isotopic masses of m/z = 355 (base peak) and m/z = 712, which are characteristic of [Ti**ent**]^2–^ and [Ti**ent**H]^–^ complexes, respectively. The experimental spectrum in positive mode was in agreement with the theoretical spectrum and contained isotopic mass of m/z = 714.1, which is characteristic of [Ti**ent**H3]^+^, representative of a salicylate form. The absence of an isotopic mass of m/z = 668 corresponding to free enterobactin (**ent**H_5_)^-^ in solution is in agreement with the 1:1 stoichiometry of the titanium–enterobactin complex. It is worth noticing that mass spectrometry did not show the formation of bi-nuclear titanium complexes or complexes containing several molecules of enterobactin, underlining the high chelating power of enterobactin.

### 3.2. Solubilization of TiO_2_-NPs by Enterobactin

**Figure 2:**
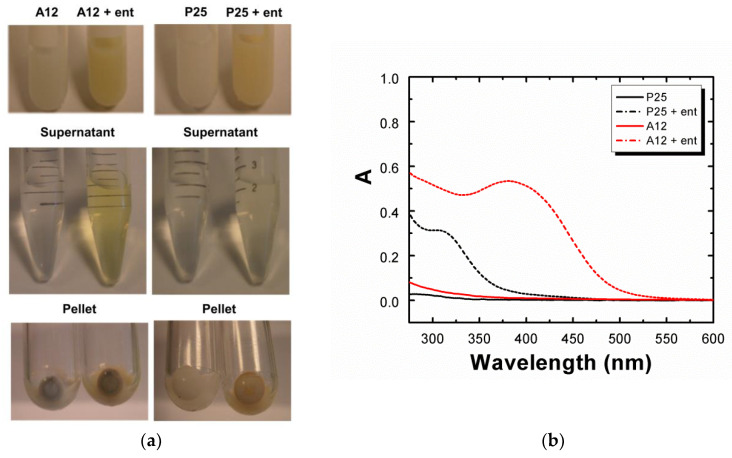
(**a**) Incubation of TiO_2_-A12 and P25 NPs at 5 mg/mL in a PP medium in the absence or presence of 50 µM enterobactin (**ent**) after 24 h at 37 °C. The supernatant (SN) and the pellets were then separated after an additional ultracentrifugation step at 75,000 rpm for 45 min at 5 °C. (**b**) UV–visible spectroscopy of the supernatants. A = absorbance.

We then investigated the solubilization of Ti(IV) from TiO_2_-NPs by enterobactin. A first set of experiments was conducted with well-characterized TiO_2_-NPs: the homemade “TiO_2_-A12” nanopowder synthesized by laser pyrolysis [38,39,40] and commercially available “Degussa P25” TiO_2_. Prior to incubation with enterobactin, TiO_2_-NPs were dispersed in water (10 mg/mL) and sonicated for 1 h in a cup-horn system (see Materials and Methods). After dilution in PP medium, the suspensions of nanoparticles (5 mg/mL) were incubated in the presence or absence of enterobactin (50 μM) for 24 h at 37 °C. After incubation and ultracentrifugation, the supernatants and the pellets were separated and collected for analysis (Figure 2a). Experimentally, we observed a rapid coloration of the solution in yellow after addition of enterobactin in the presence of TiO_2_-A12 NPs, characteristic of the titanium–enterobactin complex formation, as observed with Ti(IV) salts. The UV–visible spectrum of this supernatant showed an identical spectrum to those of the titanium–enterobactin complexes previously observed with Ti(IV) salts, with the presence of a band centered at 386 nm (OD compatible with 45 µM Ti–**ent** complex, depending on the baseline and using ε_386nm_ = 11,080 L.mol^−1^.cm^−1^), attributed above to an LMCT transition resulting from complexation of Ti(IV) with enterobactin (Figure 2b). The signature of the 1:1 stoichiometry complex is also observed by ESI-MS performed on the supernatant (Figure S3 in Supplementary Materials). The P25 incubation supernatant, however, presented only a very slight yellow coloration and the UV–visible spectrum exhibited one main band centered at 325 nm, suggesting the presence of free enterobactin.

The amount of titanium and enterobactin in the supernatant was quantified with more accuracy, respectively by ICP-AES and the Arnow method [41,42], based on the color change of enterobactin in a basic medium in the presence of sodium molybdate (see Materials and Methods). ICP-AES measurements in the absence of enterobactin showed that TiO_2_-A12 NPs intrinsically contained labile titanium ([Ti]_0_ up to 4.3 µM) whereas P25 did not. However, the amount of titanium found in the supernatant significantly increased when the nanoparticles were incubated in the presence of 50 µM enterobactin with [Ti]_ent_ = 33.0 ± 1.6 µM for TiO_2_-A12 and only [Ti]_ent_ = 3.9 ± 0.2 µM for P25. This amount, assigned to solubilized titanium from the surface of the nanoparticles by enterobactin, was much greater with TiO_2_-A12 than P25 NPs. Quantification of enterobactin‘s catechol groups by the Arnow method (Figure S4) revealed an enterobactin concentration, respectively, of 30 µM and 3.8 µM for TiO_2_-A12 and P25 NPs incubation supernatants; i.e., nearly the same amount than those of titanium quantified by ICP-AES, highlighting the 1:1 stoichiometry of the Ti–**ent** complex. Thus, ca. 60% and 7.6% of the enterobactin was complexed with Ti(IV) ions from TiO_2_-A12 and P25, respectively. From these data, we can assume that the remaining enterobactin (not quantified by the Arnow method) was absorbed on the surface of the nanoparticles and cannot be detectable by the Arnow method. This assumption could also explain the brighter orange coloration of the pellet collected with P25 than with TiO_2_-A12 NPs. In order to ascertain the difference in reactivity between the two types of TiO_2_-NPs, DLS and Zeta potential measurements were performed on the suspensions. Results indicated the presence of aggregates measuring hundreds of nanometers in size in both NPs suspension (d = 149.8 ± 25 nm for TiO_2_-A12 NPs, d = 205.2 ± 26 nm for P25 NPs) and an equivalent negative surface potential charge (ζ = -55 ± 11 mV), in accordance with **ent** and pyrophosphate ions absorbed onto the NP surface. Additional studies were conducted on annealed TiO_2_-A12 NPs; i.e., heated NPs to remove carbon impurities or ad-atoms. As ESI-MS and UV–visible spectra carried out on the supernatants of TiO_2_-A12 and annealed TiO_2_-A12 were identical (Figure S5 in Supplementary Materials), we concluded that synthesis impurities or ad-atoms were not involved in the process of solubilization of titanium by enterobactin. Thus, these parameters (ad-atoms, impurities, aggregate size, or surface charge) could not explain the large differences in amounts of solubilized titanium observed between TiO_2_-A12 (12 nm, 95% anatase) and P25 (24 nm, 85% anatase, 14% rutile). It can therefore be assumed that the binding of the enterobactin on the NP surface could depend either on their primary size and/or their crystalline structure.

This hypothesis was investigated by incubating NPs of various crystallographic forms and sizes. Their composition, size dispersion, and the amount of titanium present in the incubation supernatant quantified by ICP-AES are reported in Table 1. UV–visible spectra recorded from the incubation SNs can be found in Figure S6. Pure anatase NPs present an unambiguous spectroscopic signature corresponding to the Ti–**ent** complex (LMCT band at 386 nm) and a higher amount of Ti in the SN after incubation with **ent**. In contrast, NPs containing pure or partly rutile forms showed very small amounts of Ti in the SN (close to the limit of detection). However, pure rutile NPs used in this study possess a hydrophilic and hydrophobic coating made of Al(III) and Si(IV) that could prevent the adsorption of **ent** onto the surface and the solubilization reaction to take place. Considering the wide size dispersions observed between the different set of nanoparticles, no direct correlation between NP primary size and effective solubilization can be established. Our data suggest that the solubilization of the NPs rather involves the anatase fraction. Indeed, TiO_2_-A12 (95% anatase) and TiO_2_-R12 (85% rutile) share the same elaboration process, shape, primary and aggregate sizes [43], but exhibit a different reactivity toward **ent** (solubilized Ti: 34µM from TiO_2_-A12 and only around 4µM for TiO_2_-R12).

### 3.3. Influence of Concentration and Incubation Time on the Solubilization of TiO_2_-NPs

**Figure 3:**
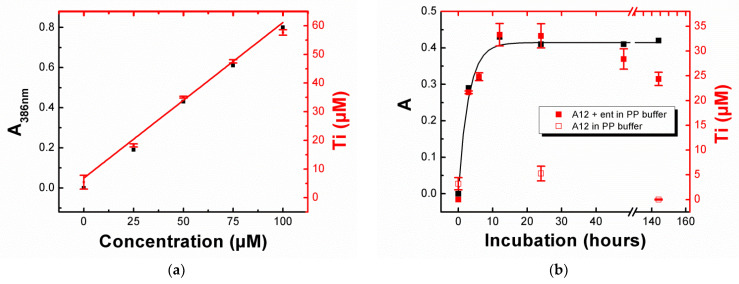
The influence of **ent** concentration and incubation time on the solubilization of TiO_2_-A12 NPs (5 mg/mL) in PP medium. UV–visible absorbance at 386 nm (black) and Ti quantified by ICP-AES (red) as a function of (**a**) **ent** concentration after 24 h incubation, and (**b**) incubation time with **ent** (50 μM). Error bars were determined by calculating the standard deviation of duplicate experiments. Red squares correspond to A12 incubation in the PP buffer in the presence (filled square) or absence of **ent** (open square).

The influence of enterobactin concentration on the solubilization rate of TiO_2_-NPs was determined by incubating TiO_2_-A12 NPs in a PP medium (5 mg/mL) for 24 h with various concentrations of enterobactin (from 0 to 100 µM). The evolution of the band at 386 nm as a function of the concentration of added enterobactin was followed by UV–visible spectroscopy (Figure S7a) and the amount of titanium in the supernatant quantified by ICP-AES (Figure 3a). Results showed that the amount of titanium dissolved in the medium varied linearly with the concentration of enterobactin and confirmed that dissolution increased in the presence of the siderophore. According to the literature [44,45], the concentration of Ti (IV) surface sites for a spherical shape anatase particle is given by the formula [Ti]_surf_ = [TiO_2_].12.5/D, where [Ti]_surf_ represents the concentration of the Ti sites on the surface, [TiO_2_] the molar concentration of TiO_2_, and D the particle diameter in Angstrom. By applying the formula to the TiO_2_-A12 NPs ([TiO_2_] = 5 mg/mL = 62.6 mM, D = 1500 Å, with the size of aggregates determined by DLS), we calculated the concentration of available titanium sites on a surface of 520 µM. By only taking into account the amount of titanium released after their incubation with enterobactin (33.0 µM), we estimated that ca. 6.3% Ti(IV) on the surface of the nanoparticles was solubilized by enterobactin at a concentration of 50 µM after 24 h of incubation.

**Figure 4:**
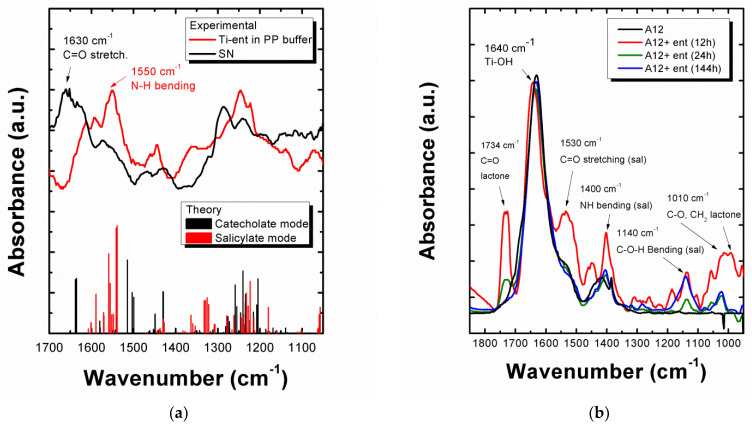
(**a**) FTIR spectra of the SN collected after 24 h of incubation at 37 °C of TiO_2_-A12 (5 mg.mL^-1^) in the presence of enterobactin (**ent**) (100 µM) in PP buffer compared to the spectra of a solution of Ti–**ent** complex (100 µM in PP medium). The peaks were assigned according to theoretical calculations of Ti–**ent** complexes in salicylate and catecholate modes (see Supplementary Materials, Section A). (**b**) FTIR spectra of the powders obtained after incubation of TiO_2_-A12 with **ent** (50 µM) at different incubation times. The spectra were normalized from the TiO_2_-specific peak at 1640 cm^-1^ (νTi–OH).

In the same manner, the evolution of TiO_2_ NP dissolution as function of the incubation time (from 0 to 144 h) was followed by UV–visible spectroscopy (Figure S7b) and ICP-AES performed on the supernatant after different incubation time of TiO_2_-A12 NPs with enterobactin (50 µM) in PP medium (5 mg/mL). Figure 3b shows that the maximum dissolution was reached after an incubation of 12 h. Contrary to the absorbance data in which a plateau was reached, we observed in ICP-AES a slight decrease in titanium after 24 h. This evolution was also observed on the TiO_2_-A12 NPs incubated without enterobactin (unfilled squares in Figure 4d). Multiple hypothesis could explain this decrease in titanium over time as the adsorption of non-complexed titanium ions on the vial, the redeposition of Ti atoms onto the NPs surface, and/or the uptake of complexed titanium by developing bacteria in the iron-depleted medium during incubation in the dark. The ability for bacteria to take up solubilized Ti in these conditions will be further investigated in Section 3.7.

### 3.4. Investigation of the Adsorption and Desorption Modes of Enterobactin on the TiO_2_-NP Surface by FTIR Spectroscopy

FTIR analysis of the SN and pellet samples was carried out to elucidate the binding mode of **ent** to Ti(IV) sites on the NP surface and determine the mechanism underlying TiO_2_-NP surface dissolution. In order to assign each vibrational band, we computed theoretical vibrational spectra using harmonic frequency calculations on DFT-optimized structures of potentially involved species: apo–**ent** or Ti–**ent** complexes in the salicylate or catecholate-binding modes (methodology, spectra, and assigned relevant modes are detailed in Supplementary Materials, Section A). Figure 4a presents the FTIR spectra recorded in the 1700–1000 cm^−1^ range on a solution of Ti–**ent** complexes synthesized in situ in the PP buffer (100 µM, pH 7.5) and on the SN collected after incubation of TiO_2_-A12 NPs (5 mg/mL) with **ent** (100 µM). The experimental peaks were in good agreement with peaks obtained from simulation and consistent with those described by Upritchard et al. [35] concerning the spectra of **ent** and its adsorption onto TiO_2_ films. However, the spectra presented major differences between 1700–1500 cm^−1^, where markers of the two coordination modes were found according to calculations (bars in Figure 4a). In this range, vibrational bands observed in the Ti–**ent** complex spectrum fitted well with those of the simulated spectrum of the Ti–**ent** salicylate complex. Markers of this form were found experimentally at 1530, 1550, and 1614 cm^−1^, corresponding to different vibrational modes of the C=O (amide I, lactone), N-H (amide II), and C–C (catechol rings) bonds, respectively. On the contrary, the SN spectrum showed a series of peaks at 1660, 1651, and 1634 cm^−1^, corresponding to the stretching of the C=O bonds (amide I) calculated in the catecholate mode. Thus, although complexation with partially protonated **ent** appears to be the most stable form in this medium and pH range, interaction between **ent** and the TiO_2_-NP favored the formation of the catecholate complex (deprotonated **ent**). FTIR spectra of the powder confirmed the presence of **ent** bound to the surface (Figure 4b). The progressive disappearance of the characteristic peaks of **ent** as incubation time increased (especially the peak at 1730 cm^−1^, assigned to C=O bonds of the lactone backbone), reveals it progressive desorption. Potential markers of the salicylate mode were observed (exp. peaks at 1530 cm^−1^ and 1400 cm^−1^, possibly attributable to C=O stretching (th. 1430 cm^−1^) and N-H bending (th. 1327 cm^−1^); exp. peak at 1140 cm^−1^, corresponding to C–O–H bending of the catechols in salicylate mode (th. 1180 cm^−1^). Differences in band wavenumber could be imputable to the conformational restrictions of **ent**, coordinating to a surface versus metal ions in solution. Contrary to Uprichard et al. [35], the experimental spectrum of the pellet is closest to the theoretical spectrum of the salicylate mode compared to the catecholate but it is not possible to assert the adsorption mode of **ent** on the surface as markers are hidden by the broad peak at 1638 cm^−1^ characteristic of Ti–OH bonds from the NP hydrated surface. After 144 h of incubation with **ent**, the only remaining characteristic peaks of TiO_2_ (1640 cm^−1^: Ti–OH vibration; 1400 cm^−1^: O (TiO_2_)_-O (H_2_O) lattice vibration [33]) and the bands at 1140 and 1027 cm^−1^ were associated to C–O–H bending and C–O stretching from catechol groups, respectively. The disappearance of the triserine lactone markers (peaks at 1734 and 1010 cm^−1^) suggest desorption or a degradation of the **ent** edifice via the breakage of the triserine lactone. The full integrity of enterobactin absorbed onto the surface and its partial hydrolysis were previously questioned by Upritchard et al. [35].

### 3.5. XPS Surface Analysis of the TiO_2_-NPs Interaction with Enterobactin

**Figure 5:**
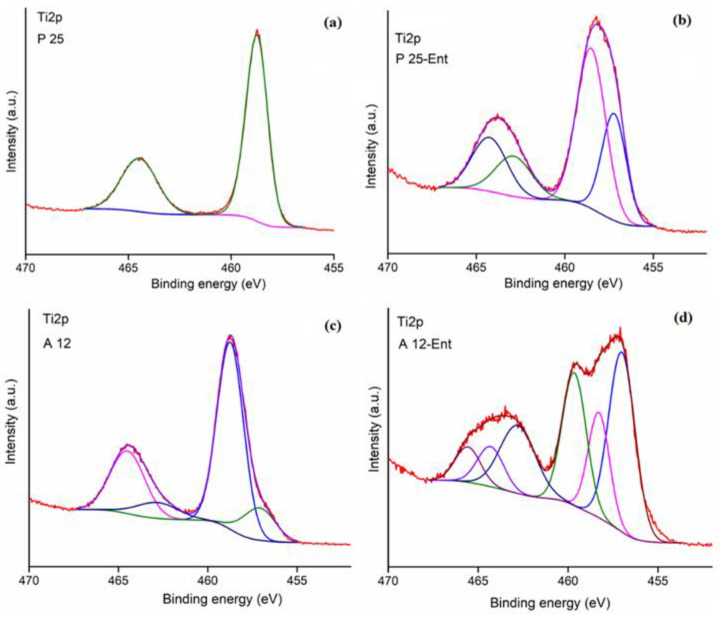
High-resolution XPS spectra of the Ti2p peaks of the P25 (**a**,**b**) and A12 (**c**,**d**) samples before (**a**,**c**) and after (**b**,**d**) incubation with **ent** for 24 h.

The elemental composition and chemical states of the A12 and P25 samples before and after incubation with enterobactin for 24 h were evaluated by XPS allowing us to analyze the surface of the titanium powders (see Supplementary Materials, Section B for detailed of the analysis). The XPS survey spectra of the samples (Supplementary Figure S13) mainly revealed prominent peaks of carbon (C1s) (Figure S14), oxygen (O1s) (Figure S15), and titanium (Ti2p) (Figure S16), as well as small N1s (Figure S17), P2p (Figure S18), and Cl2p contributions. Ti^4+^ but also some Ti^3+^ signatures are present in the high-resolution XPS spectra of the Ti2p peaks (Figure 5). The Ti^3+^ contribution increased in intensity after incubation of both P25 and A12 with **ent** for 24 h, which is related to surface defects due to the presence of oxygen vacancies. For A12 incubated with **ent**, the Ti–OH signature was observed in high-resolution XPS spectra of the O1s peaks. Furthermore, nitrogen and phosphorus elements (P5+ oxidation state in PO_4_^2−/3−^) are present in the A12 and P25 samples before and after incubation with **ent** for 24h, and some chlore in P25, all probably due to surface contamination during the process of obtaining the TiO_2_ powders (see Supplementary Materials, Section B for details of the analysis).

### 3.6. Application to E171 Food Additives

As mentioned in the introduction, titanium dioxide is widely found and used in the food industry as additives (E171) for its whiteness, brightness, and opacifying power. To apply our observations to an everyday-life case study, similar experiments were performed on two commercially available batches of E171 additives (found predominantly in the anatase phase) [16,46]. DLS measurements on these two batches after dispersion in a cup-horn system (Table 1) showed a high ratio of particles with a size inferior to 100 nm (>50% in number for batch 2). Therefore, we assigned these commercial products to the category of nanomaterials according to the definition of the European Commission (EU, 2011, Commission Recommendation of 18 october 2011 (2011/696/EU).O.J.L. 275:38-40). Table 1 reports the various populations observed in both batches and the amount of titanium quantified by ICP-AES in the supernatant before and after incubation of additives E171 (5 mg/mL) with enterobactin (50 µM) in a PP medium. In the absence of enterobactin, an important amount of labile titanium was found ([Ti]_0_ = 10-11 µM). Incubation with enterobactin (50 µM) for 24 h at 37 °C led to an increase in the amount of titanium found in the supernatant ([Ti]_ent_ = [Ti]_0_ +3-4 µM) due to the enterobactin-promoted dissolution of TiO_2_ particles present in the additives.

### 3.7. Evidence of a Ti–Enterobactin Complex Entrance in Bacteria

We provide direct evidence of solubilization in a biological medium of titanium from TiO_2_-NPs by enterobactin, the main bacterial siderophore. Although the solubilized titanium concentration represents only a small percentage of the titanium on the surface of TiO_2_-NPs, it is, however, not negligible compared to the micromolar iron concentration in bacteria [21]. Finally, the possible entrance of the Ti–**ent** complex in the bacteria via the Fe–enterobactin complex route was investigated. Siderophores, and the specific machinery necessary for their import after complexation with iron, are produced specifically in response to iron deficiency [47]. ICP-AES was performed to measure the titanium content of *Escherichia coli* (BL21 strain) after growing in the presence of a Ti–enterobactin complex (3.5 µM, with regard to the average micromolar release of Ti(IV) observed in our exposure conditions to TiO_2_-NPs) in rich (LB) or iron-depleted medium (PP). Titanium was not detectable in bacteria growing in a rich medium, nor in a control without the Ti–enterobactin complex, but, interestingly, a titanium concentration of 36 µM ± 5.6 (SEM) was measured in bacteria growing in an iron-depleted medium. These results prove that the Ti–enterobactin complex can be transported and accumulates in bacteria, presumably using the iron-uptake machinery that is inducible in iron-deficiency conditions.

## 4. Discussion

### 4.1. Influence of the Crystallographic Form and Primary Size of TiO_2_-NPs on Their Dissolution Rate

In its mineral form, TiO_2_ commonly exists in four polymorphic structures: anatase, rutile, brookite, and Akaogiite (although very rare) [2,48]. In these structures, titanium atoms are placed in an octahedral environment composed of oxygen [49]. The density of the crystal and the length of the Ti-O bonds within this octahedron vary among these polymorphic structures, which have distinct physicochemical properties and reactivities at the crystal surface [1,2]. Our data suggest that the solubilization of the NPs involves the anatase fraction of the crystallographic form. This is clearly illustrated by the results obtained with TiO_2_-A12 (95% anatase) and TiO_2_-R12 (85% rutile), which are NPs that share the same elaboration process, primary size, and aggregate size, and which exhibit a different reactivity toward enterobactin (solubilized titanium: 34 µM from TiO_2_-A12 and only around 4 µM for TiO_2_-R12). The different arrangement of atoms at the surface on both structures (anatase, rutile) leads to the favorable positioning of the chelating ligand to be absorbed onto the surface and to detach one titanium atom. The anatase polymorph is around 9% less dense and around 1.2 kcal/mol less stable than rutile [50,51]. Surface properties are also of major importance and must be considered in our case to explain the highest amount of titanium solubilized, especially in the case of TiO_2_-A12 NP. Although the (101) surface is the most exposed surface in an anatase lattice [50,52], the under tensile stress (001) surface presents the highest reactivity due to the unnatural geometrical configuration of the bridging oxygen shortening the Ti-O bonds and closing the Ti-O-Ti angles [53,54]. Another key aspect of the reactivity of the Ti(IV) sites relies on the presence of defects on the NPs surface. Reactions on active sites located on crystalline defects, such as oxygen vacancies, steps, kinks, and edges of TiO_2_, are most likely to occur because of their lower activation energies, promoting the surface reactions to take place [52,55]. This aspect is nicely illustrated by the highest reactivity observed in the case of TiO_2_-A12 NPs. Indeed, the main characteristic of these NPs compared to the others is the light-blue color of the powder. This color was associated to the presence of sub-oxide TiO_2−x_ (with x < 2), and particularly of Ti_2_O_3_ formed by a reduction in oxide during laser pyrolysis in a reductive atmosphere [56,57]. This was confirmed by the XPS data of the high-resolution spectra of the Ti2p peaks analysis that showed an increase in the proportion of Ti^3+^, known to increase in the presence of surface defects due to oxygen vacancies (Figure S16 and detailed analysis in Supplementary Materials, Section B). The partially reductive surface of the NPs become a reactive site since it possesses the highest affinity toward electron-donor species such as enterobactin. The triggering of redox reactions via the absorption of enterobactin can then weaken the Ti-O bonds and lead to the departure of a Ti ion from the surface.

As illustrated by the FTIR analysis, different coordination modes of enterobactin on the surface are possible: a monodentate O binding from the catechol moiety on a Ti(IV) site, a bidentate complexation mode on two adjacent Ti(IV), and/or a chelated mode where the two OH groups from the catechol are bound to one Ti(IV) site [58,59]. Interestingly, monodentate absorption of catechols preferentially occurred (both in theoretical and experimental studies) on the (100) crystallographic plan of the rutile lattice [60] while bidentate and chelated adsorption is favored on an (101) and (001) anatase surface [61,62]. These differences could explain the reactivity and dissolution observed with anatase forms, as conformation on the surface favors the chelated mode.

### 4.2. Surface Solubilization Mechanism

Based on the literature concerning the dissolution of minerals by siderophores and other chelating biomolecules [22,63,64,65,66,67], the mechanism of solubilization of Ti(IV) atoms from the surface could include three steps: (i) the adsorption of the ligands at the surface sites by ligand exchange reaction with hydroxyl surface groups, where they polarize and weaken the metal–oxygen bonds, (ii) the rate-limiting detachment of the reacting surface metal species, promoted by strong chelation; and (iii) the transport of the detached metal complex into the bulk solution followed by the fast protonation reactions of the oxygens at the initial active site to restore the surface [22,68]. Concerning the protonation reaction (iii), Li et al. [59] reported that both modes (monodentate and bidentate) could simultaneously exist and can switch from one to the other via proton exchange between the surface and the absorbed catechol. 

**Figure 6:**
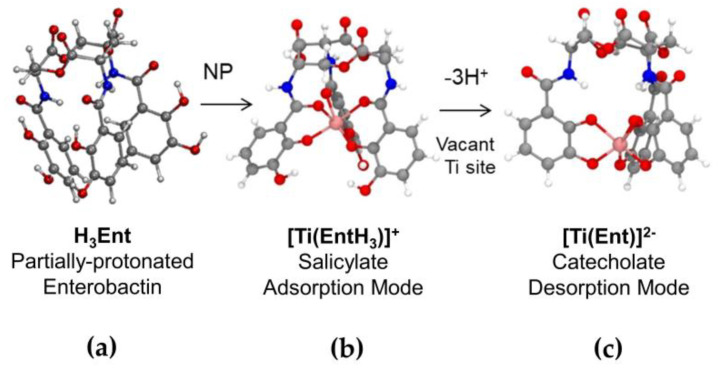
Surface solubilization mechanism of the TiO_2_-NPs by enterobactin. The mechanism consists of (**a**) the approach of a partially protonated enterobactin near neutral pH toward the TiO_2_ NP surface; (**b**) the binding to a Ti site in the salicylate mode via one or two bidentate catechols group(s) first, and then its full chelation; and (**c**) the rate-limiting detachment of the Ti atom, leading to the release of a catecholate Ti-**ent** complex (1:1 stoichiometry) in the environment, followed by a proton-exchange reaction with the vacant site to regenerate the surface.

Regarding our dataset, we propose the following mechanism, illustrated in Figure 6, to account for the dissolution of TiO_2_-NPs by the enterobactin siderophore in a biological medium. The rate-limiting detachment of the Ti(IV) atom is first favored by the adsorption on Ti sites of partially protonated **ent** at this pH range (supported by ESI-MS and FTIR observation) in a chelating mode via one or two of its catechol units, polarizing and weakening the Ti-O bonds. Indeed, the direct adsorption via the three catechols units is very unlikely due to steric hindrance and will occur via one or two catechol units at first, and then eventually the chelation occurs via the third catechol to detach the Ti from the surface. As mentioned above, the structure and organization of the Ti units on the surface plays an important role as we could imagine **ent** to bind two Ti sites instead of one in rutile surface as the latter is denser than the anatase surface, as described in the literature [60]. The two-site adsorption could prevent the chelation and solubilization of the Ti from the surface, explaining the lowest solubilization rate observed with the rutile TiO_2_ particles. This adsorption occurs in the salicylate mode, preferentially on defects sites or on the (001) and (101) planes of an anatase lattice. The release of a Ti-**ent** complex in a 1:1 stoichiometry and catecholate mode suggest the loss of the **ent** protons during interaction with the TiO_2_ surface. After removal of the Ti ion from the surface, a proton transfer reaction occurs from the partially protonated enterobactin to the vacant site (uncoordinated oxygens), in order to maintain the equilibrium of charges at the site of action. After the proton transfer reaction from **ent** to the active site, the Ti–**ent** complex is then progressively released into the medium where it can be eventually taken up by bacteria.

The dissolution process proposed for enterobactin could be complicated by steric factors for larger biomolecules such as desferrioxamine B (DFOB), which is highly flexible due to the presence of two alkane chains between each hydroxamate group. Recently, it has been shown that desferrioxamine B is able to bind Ti(IV) and form an hexacoordinated Ti-DFOB complex [69]. Thus, further similar experiments with other siderophores, such as DFOB, are required to investigate in more detail the influence of the architecture and denticity of the ligand on the dissolution process.

### 4.3. Results Placed in the Context of TiO_2_ NP Ingestion and Iron Homeostasis

Titanium is highly abundant and nontoxic but there is still a debate surrounding the possible toxicity of its nanoparticulate form, in particular in the case of long-term exposure through ingestion. Very few studies have focused on the effect of titanium dioxide nanoparticles on the microbiota and mucus. Results from Chen H. et al. [70] indicated that a 7-day intake of TiO_2_-NPs by mice at levels consistent with general public daily exposure levels induces no strong perturbation of gut microbiota composition. The normal gut microbiota imparts a specific function in host nutrient metabolism, xenobiotic and drug metabolism, maintenance of structural integrity of the gut mucosal barrier, immunomodulation, and protection against pathogens [71]. Microbiota have also been recently described to be implicated in iron homeostasis [72], and the presence of a Ti-enterobactin complex in the digestive tract could perturb this equilibrium. Indeed, there is a tight competition between bacteria in the microbiota for any available iron [57] in the environment and, as mentioned in introduction, enterobactin has a better affinity for titanium than iron [30,31]. Thus, the enterobactins sequestered by Ti ions (present initially in the additives or released from dissolution) or absorbed onto the NP surface become unavailable to transport iron inside the bacteria. In this article, we demonstrate that Ti(IV) enters the bacteria. This might perturb other iron-based pathways, leading to a disruption in the iron homeostasis of the microbiota. Bacterial cells require a cytoplasmic iron concentration of 10^−6^ M for growth [57]; i.e., in the range of titanium solubilized by enterobactin, determined in our study for the whole set of anatase NPs used. As a consequence, the presence of titanium in the micromolar range could potentially alter iron homeostasis. Beside the bacteria, the Ti-**ent** complexes may have various fates in the organism. For example, Ti-**ent** complexes could interact with siderocalins, proteins of the immune system, secreted from some host cells in response to bacterial siderophores as a defense mechanism against pathogenic microorganisms, which works by trapping the siderophore–iron complexes and making the iron unavailable for bacteria [56,73,74]. In this case, siderocalins will be diverted from their important role as immunity defense. Interestingly, it has been shown recently that the microbiota of mice lacking siderocalin is modified and is more prone to intestinal inflammatory disease. Siderocalins are critical for intestinal homeostasis [74]. Ti-**ent** complexes trapped by siderocalins could also be potentially transported into gut cells via the siderocalin pathway [75], leading to the presence of titanium in the bloodstream. It could then be transported by transferrin (an iron-binding blood plasma glycoprotein) for which Ti(IV) has a higher affinity than iron [30]. Finally, titanium could be distributed to other cells of the human body and may replace iron in Fe proteins or ferritin [76] and thus perturb important functions in the cells. Assuming that titanium could accumulate within cells and disrupt iron homeostasis, the potential toxicity resulting from long-term exposure to E171 should be considered, in particular in pathological conditions. Our hypothesis could explain the toxicity of E171 in some animal model studies, as it was shown to be linked with immune system perturbation.

## 5. Conclusions

In this study, we provide direct evidence of the surface solubilization in a biological medium of titanium from TiO_2_-NPs by enterobactin, the main bacterial siderophore. Although the solubilized titanium concentration represents only a small percentage of the titanium on the surface of TiO_2_-NPs (ca. 6.3% of Ti atoms on the surface for the most reactive A12 NPs occurring within 24 h), it is, however, not negligible compared to the micromolar iron concentration needed in bacteria. We show that enterobactin is able to bind to the surface of the nanoparticles, either anatase or rutile, and to remove reactive titanium (preferably from an anatase surface) using the ligand-promoted mechanism reported in the literature, which explains the dissolution of other metal oxides and minerals by natural chelating ligands. In this mechanism, enterobactin binds titanium via its three catechol groups, resulting in the weakening of the Ti–O bond and the detachment of the 1:1 titanium–enterobactin complex. The crystalline form and the agglomerate size of the TiO_2_-NPs play key roles in the extent of the solubilization because of the favored chelating position and greater exposed surface for enterobactin to bind Ti(IV) sites. Given that TiO_2_-NPs are found in everyday-life products and ingested in large quantities, in particular as food additives (E171), as well as in the environment, our observations of siderophore-assisted dissolution may pinpoint a potential risk to humans through chronic exposure. It would therefore be interesting to pursue more in-depth studies of the entrance mechanisms of titanium within bacteria and eukaryotic cells, as well as its potential accumulation and possible entry via other organic chelators.

## Figures and Tables

**Table 1:** Physicochemical characteristics of the TiO_2_-NPs and their solubilization by enterobactin.

TiO_2_ NP	CAPC500 ^(3)^	101 JRC	TiO_2_-A12^(4)^	P25 ^(4)^	Sigma	TiO_2_-R12 ^(4)^	104 JRC	103 JRC	E171 Batch 1	E171 Batch 2
Shape	Sphere	Sphere	Sphere	Sphere	Sphere	Sphere	Sphere	Sphere	-	-
Primary size	3 nm	6 nm	12 nm	24 nm	<25 nm	12 nm	20 nm	20 nm	-	-
Mean size diameter after sonication (nm)	518	Aggregates	150	205	502	192	177	38	peak1: 50 (75% *) peak 2: 311 (25% *)	peak1: 38 (92% *) peak2: 255 (8% *)
% polydispersity	24	-	17	13	39	22	30	9	peak1: 9 peak2: 22	peak1: 11 peak2: 21
Crystalline composition	100% anatase	100% anatase	95% anatase	86% anatase 14% rutile	100% anatase	85% rutile 15% anatase	100% rutile hydrophilic	100% rutile hydrophobic	Mainly anatase	Mainly anatase
[Ti]_0_ (µM) ^(1)^	1.3 ± 0.1	b.d.	4.5 ± 0.2	b.d.	0.8 ± 0.2	0.4 ± 0.1	4.5 ± 0.5	3.4 ± 0.2	11.1 ± 0.6	10.1 ± 0.6
[Ti]_ent_ (µM) ^(2)^	5.8 ± 0.6	4.5 ± 0.3	33.0 ± 1.6	3.9 ± 0.2	4.1 ± 0.3	4.5 ± 0.1	4.9 ± 0.4	3.5 ± 0.1	14.6 ± 0.4	14.0 ± 0.1

The average diameter and polydispersity of the suspensions were determined by DLS measurements. Quantification of Ti solubilized from NPs (5 mg/mL) by enterobactin (**ent**) (50 µM) determined by ICP-AES. ^(1)^ [Ti]_0_: amount of Ti released by the TiO_2_-NPs without **ent** (labile Ti), ^(2)^ [Ti]_ent_: amount of Ti released after incubation of TiO_2_-NP with **ent**, b.d.: below detection limit, (*) in number. ^(3)^ Laisney et al. [33]. (4) Teulon et al. [43].

## Data Availability

The data presented in this study are available in this article and its Supplementary Material.

## Supplementary Materials

The following supplementary information including Section A (theoretical data) and B (XPS anal-ysis of the TiO_2_-NPs interaction with enterobactin) can be downloaded at: https://www.mdpi.com/article/10.3390/biom12101516/s1, as a supplementary information pdf file. Table S1: TiO_2_ nanoparticles and their main physico-chemical characteristics; Figure S1: UV-visible spectra following the metallation of enterobactin by TiCl4 in water; Figure S2: ESI-MS spectrum of the Ti-ent complex; Figure S3: ESI-MS spectra of the incubation supernatant of TiO_2_-A12 NP with enterobactin; Figure S4: Quantification of entero-bactin by the Arnow method present in the supernatants; Figure S5: UV-visible spectroscopy and mass spectra of the supernatants after incubation of enterobactin with TiO_2_-A12 Annealed and TiO_2_-A12 NP; Figure S6: UV-visible spectra of supernatants after incubation of enterobactin with anatase and rutile TiO_2_-NP; Figure S7: UV-Vis spectra of the incubation supernatants as function of the enterobactin concentration and incubation time; Figure S8: Optimized structures for totally protonated apo-enterobactin, [Ti(**ent**)]2− complex in the “catecholate” binding mode and [Ti(entH3)]+ complex in the “salicylate” binding mode; Figure S9: Simulated UV-Vis spectra; Figure S10: Simulated IR spectrum for the fully protonated enterobactin; Table S2: Attribution of peaks for the fully protonated enterobactin with corresponding experimental data; Figure S11: Simulated IR spectrum for the [Ti(entH3)]+ complex, in the “salicylate” binding mode; Table S3: Attribution of peaks for the [Ti(entH3)]+ complex in the “salicylate” binding mode, with corresponding experi-mental data; Figure S12: Simulated IR spectrum for the [Ti(**ent**)]2− complex, in the “catecholate” binding mode; Table S4: Attribution of peaks for the [Ti(**ent**)]2− complex in the “catecholate” binding mode, with corresponding experimental data; Figure S13: XPS survey spectra of the P25 and A12 samples before and after incubation with enterobactin; Figure S14: High-resolution XPS spectra of the C1s peaks; Figure S15: High-resolution XPS spectra of the O1s peaks; Figure S16: High-resolution XPS spectra of the Ti2p peaks; Figure S17: High-resolution XPS spectra of the N1s peaks; Figure S18: High-resolution XPS spectra of the P2p peaks.
