# Supplementary material for: Ligand-Promoted Surface Solubilization of TiO2 Nanoparticles by the Enterobactin Siderophore in Biological Medium
Source: Biomolecules. 2022 Oct 19;12(10):1516. doi: 10.3390/biom12101516 (PMC9599204; doi:10.3390/biom12101516)
Supplement: Supplementary file 1 [file biomolecules-12-01516-s001.zip › biomolecules-1938049-supplementary - final.pdf]

Ligand-promoted surface solubilization of TiO_2 nanoparticles by the enterobactin siderophore in biological medium

Jérôme Laisney [1,*], Mireille Chevallet [1], Caroline Fauquant [1], Camille Sageot [1], Yohann Moreau [1], Daniela Predoi [2], Nathalie Herlin-Boime [2], Colette Lebrun [3] and Isabelle Michaud-Soret [1,*]

Table of Contents:

Supplementary Materials.. **S2-S9**

Section A: Theoretical Data.. **S10-S15**

Section B: XPS analysis of the TiO_2 NPs interaction with Enterobactin.............................. **S16-S24**

Supplementary Materials

Table S1. TiO_2 nanoparticles and their main physicochemical characteristics.

Sample name	Supplier	Batch number	Size (mean diameter)	Crystalline composition
CAPC50= CristalACTiV™ PC500 [1]	Tronox		3 nm	100% anatase
TiO_2-A12 [2]	Home made	Octi 50	12 nm	95% anatase
TiO_2-A12 annealed	Home made	Octi 79R	18 nm	99.7% anatase
TiO_2-R12	Home made	Octi 195R	12 nm	85% rutile<br>15% anatase
Sigma TiO_2	Aldrich 637-254	MKBB1944	< 25 nm	100% anatase
P25	Degussa		24 nm	86% anatase<br>14% rutile
101JRC	European Joint Research Center (JRC)	n ident: 1117	6 nm	100% anatase
103JRC	JRC	n ident: 0684	20 nm	100% rutile with hydrophobic coating (alumine+glycerine)
104JRC	JRC	n ident: 0686	20 nm	100% rutile with hydrophilic coating (alumine+diméthycone)

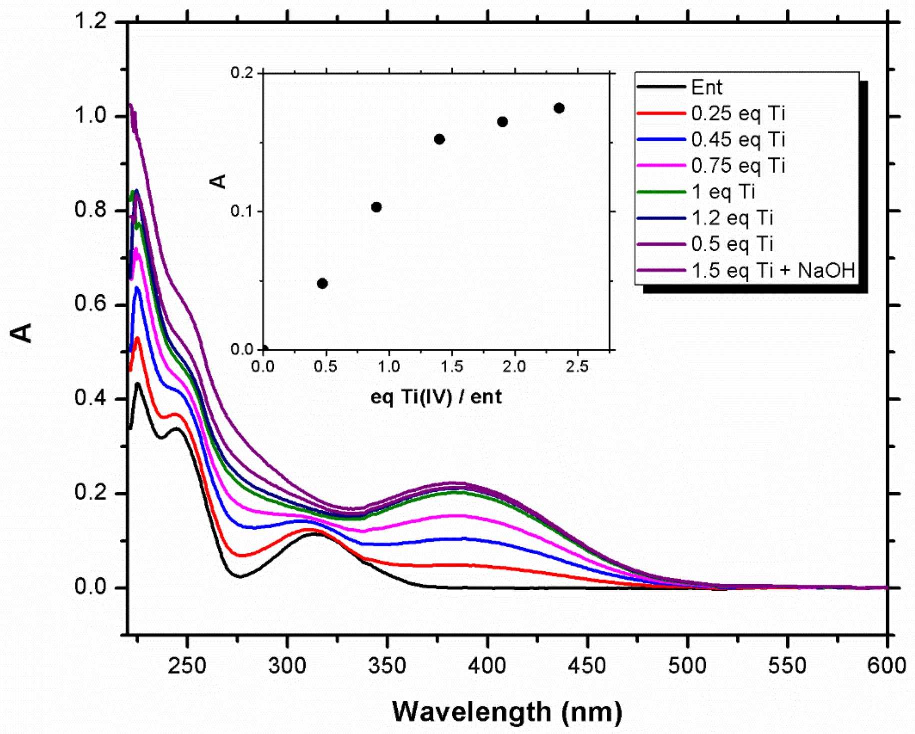

Figure S1. UV-visible spectroscopy showing the metallation of enterobactin (**ent**) (12 μM) by successive additions of equivalents of Ti(IV) ($TiCl_4$) in water. (Inserts) Determination of Ti-enterobactin complex stoichiometry in water according to the evolution of the absorbance at 386 nm during addition of Ti(IV). A= absorbance.

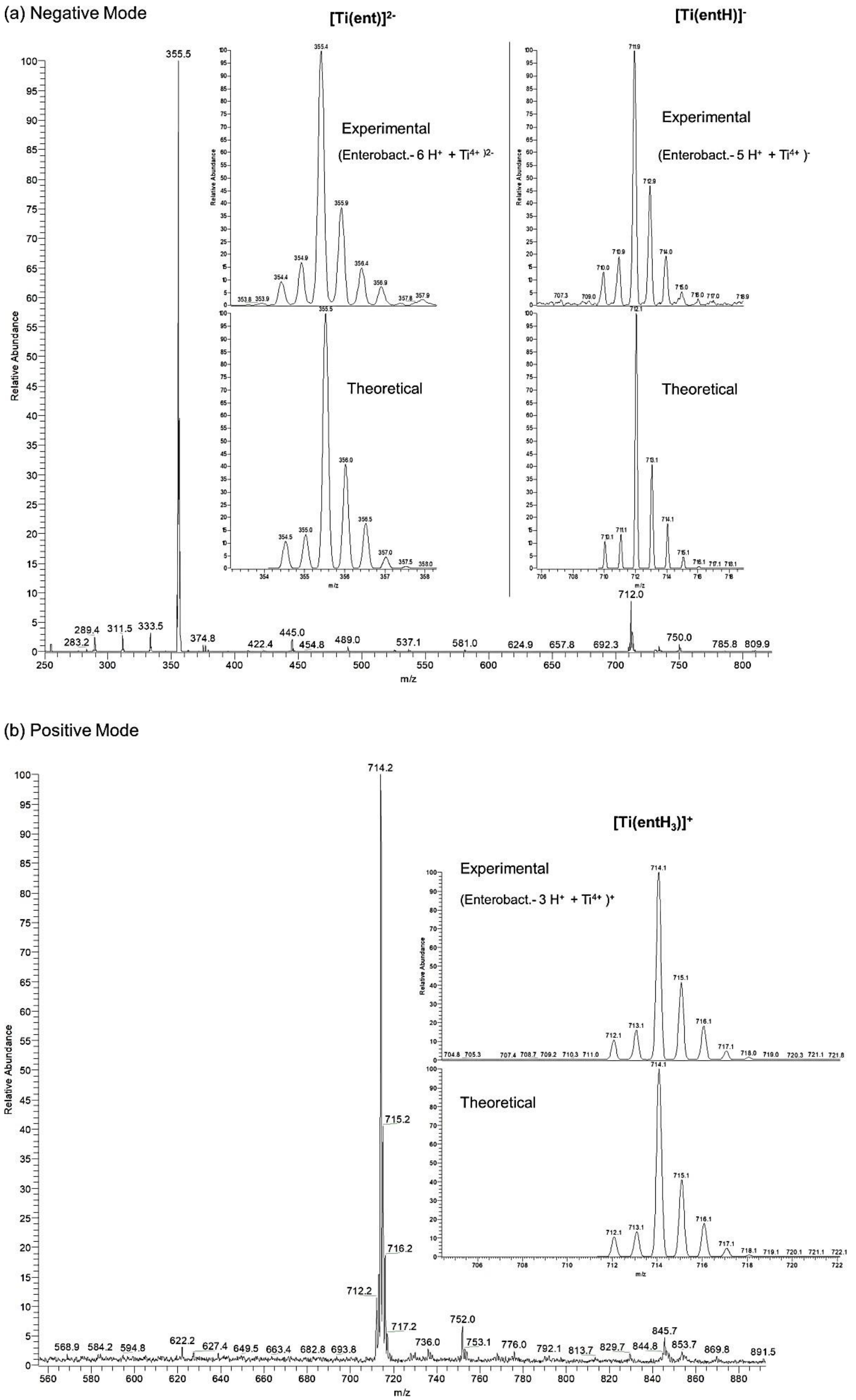

Figure S2. ESI-MS spectrum of the Ti-**ent** complex obtained (a) in negative mode and (b) in positive mode after incubation of enterobactin (50 μM) in presence of $TiCl_4$ (50 μM) for 1 h in water. (Insert) Isotopic distribution.

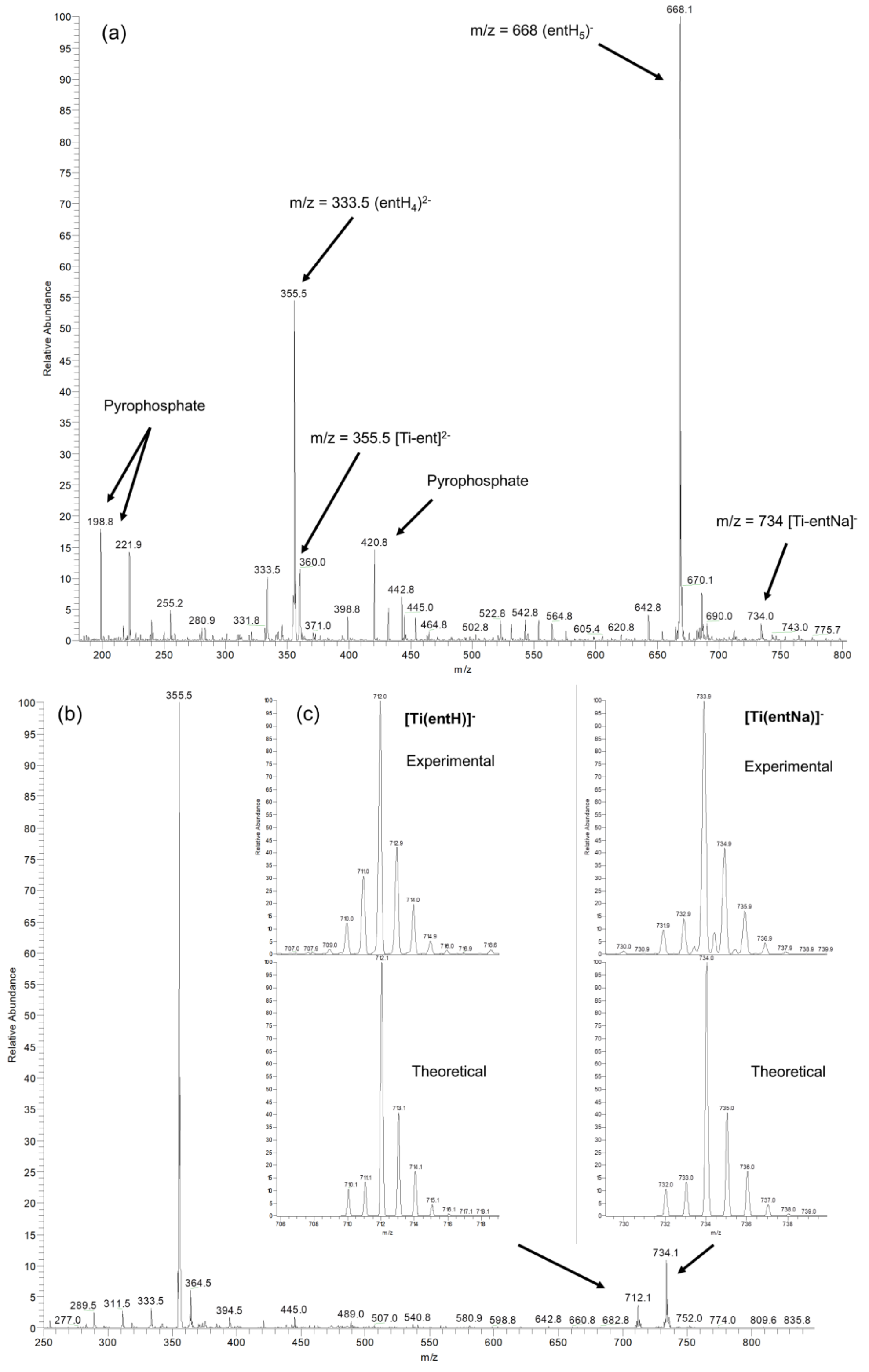

Figure S3. ESI-MS spectra obtained in negative mode on the supernatant after incubation of TiO_2-A12 NP (5 mg.mL^{-1}) with enterobactin (**ent**) (50 µM) at 37°C for 5 min (a) and 60 h (b). The supernatant were re-suspended in water containing 5% pyrophosphate. Mass at m/z = 355 corresponds to two-negatively charged enterobactin titanium complex [Ti-**ent**]$^{2-}$; m/z = 668 corresponds to free enterobactin (**ent**H5)$^{-}$, m/z = 712 corresponds to the Ti-enterobactin complex charged negatively [Ti-**ent**H]$^{-}$; m/z = 734 corresponds to the Ti-enterobactin complex [Ti-**ent**Na]$^{-}$. (c) Isotopic distribution of the mass spectrum of the Ti-enterobactin complex.

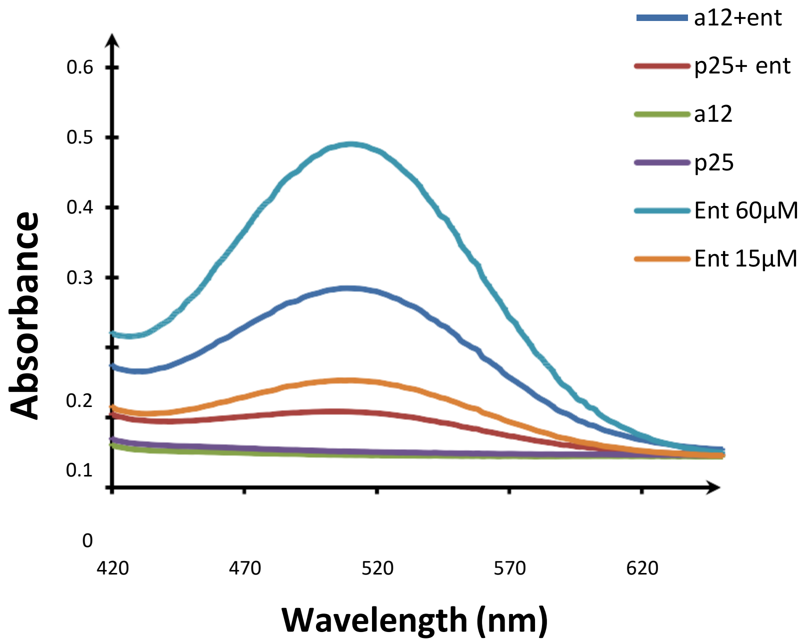

Figure S4. Quantification by the Arnow method [3] of enterobactin (**ent**) present in the supernatants after incubation of enterobactin (50 µM) with TiO_2-A12 and P25 NP (5 mg.mL^{-1}).

a)

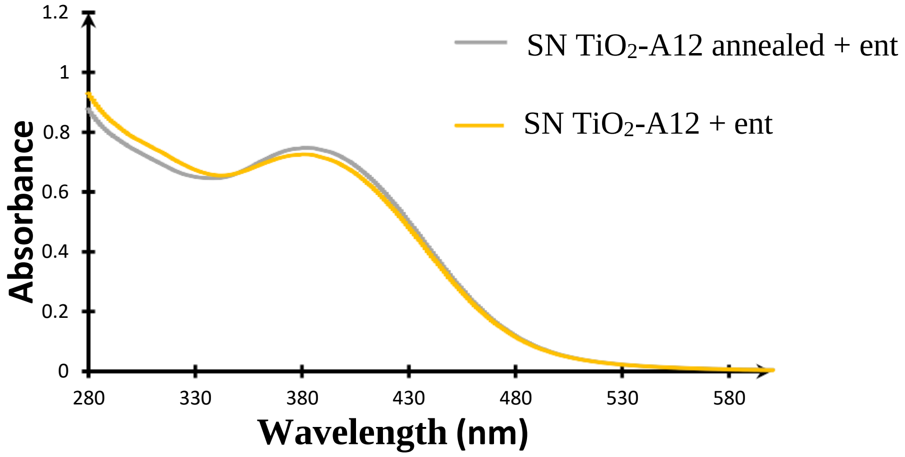

b)

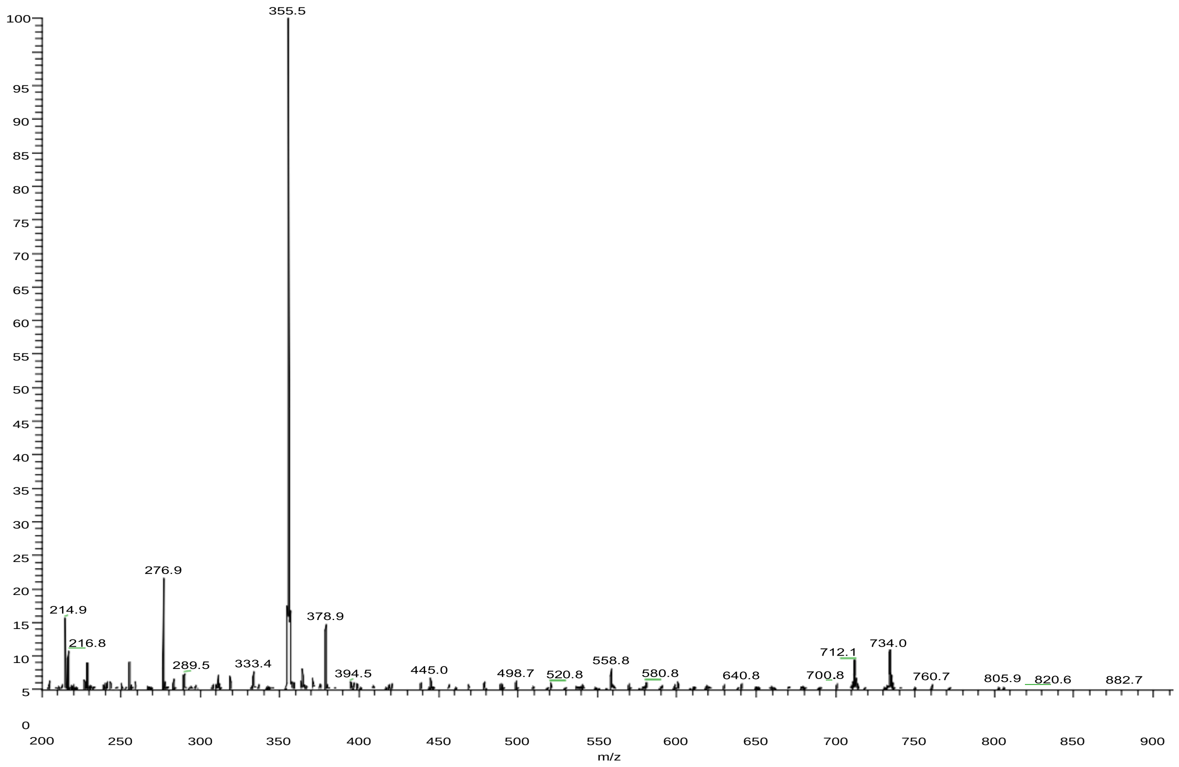

Figure S5. (a) UV-visible spectroscopy of the supernatant collected after incubation of enterobactin (**ent**) (50 µM) with TiO_2-Annealed (5 mg.mL^{-1}) and TiO_2-A12 NP (5 mg.mL^{-1}). The spectra were carried out on the supernatants after the suspensions been ultracentrifuged at 75 000 rpm for 45 min at 5°C and filtered on a 3 kDa Centricon unit. **(b)** Mass spectra obtained by ESI-MS in negative mode on the supernatant after incubation at 50°C for 50h of annealed TiO_2-A12 (5 mg.mL^{-1}) with enterobactin (50 µM). The supernatant were re-suspended in water containing 5% pyrophosphate. The mass of m/z = 355 corresponds to the two-negatively charged enterobactin titanium complex [Ti-**ent**]$^{2-}$; m/z = 712 corresponds to the titanium-enterobactin complex negatively charged [Ti-**ent**H]$^{-}$, m/z

= 734 corresponds to the titanium-enterobactin complex [Ti-**ent**Na]$^{-}$.

a)

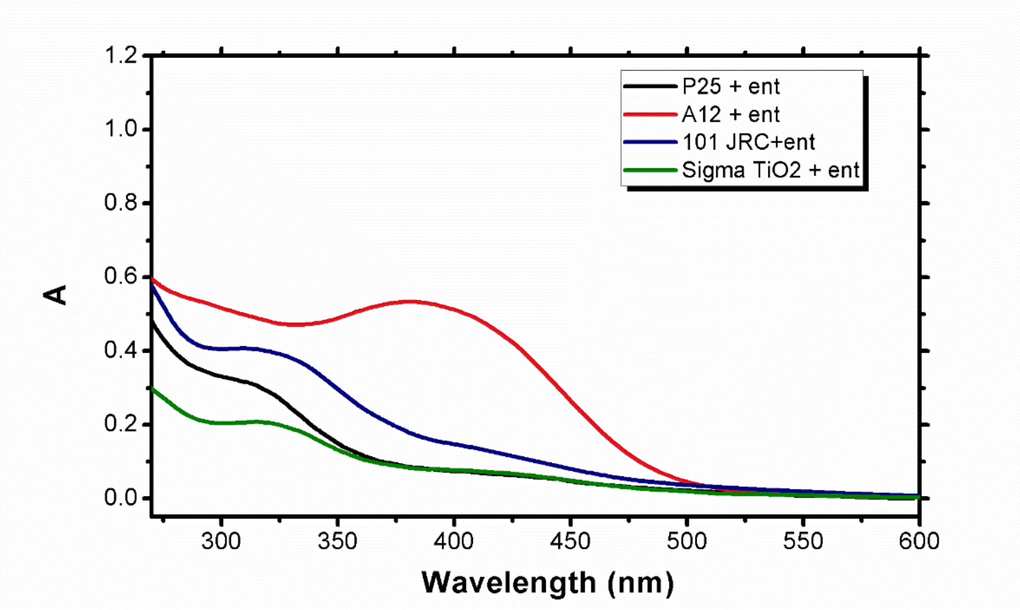

b)

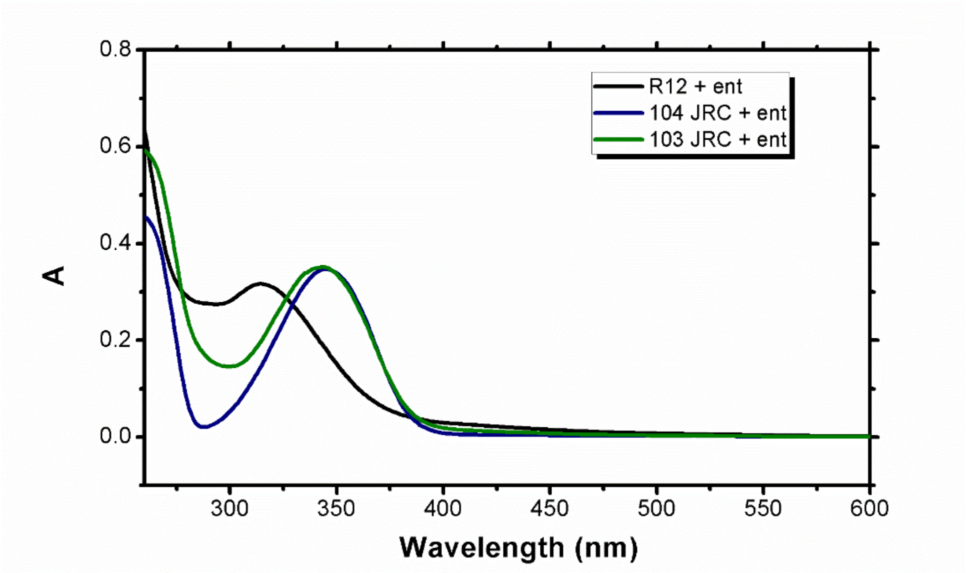

Figure S6. UV-visible spectra monitoring the metallation of enterobactin (**ent**) by Ti (IV) from mostly anatase (a) and rutile (b) TiO_2-NP. All NPs used (5 mg.mL^{-1}) were suspended in PP medium and incubated 24 h under stirring at 37°C in presence of enterobactin (50 µM). The UV-visible spectra were carried out on the supernatants. A= absorbance.

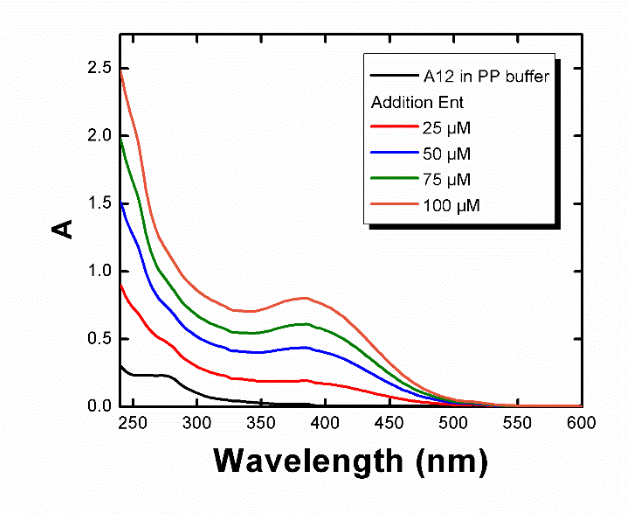

a)

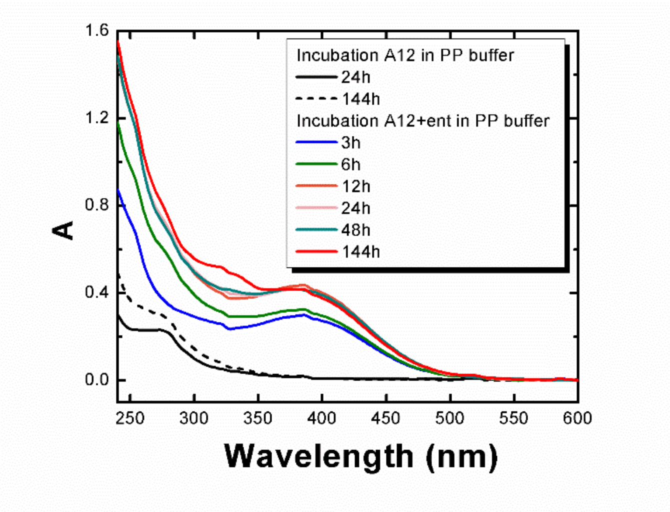

b)

Figure S7. Influence of the concentration of enterobactin (**ent**) and the incubation time on the dissolution of TiO_2-A12 NPs. (a) UV-visible spectra of the supernatants measured after a 24 h incubation at 37°C of TiO_2-A12 (5 mg.mL^{-1}) in PP buffer medium with 0 to 100 µM of enterobactin; (b) influence of the incubation times on the dissolution of TiO_2-A12 NPs (5 mg.mL^{-1}) with enterobactin (50 µM) in PP medium followed by UV-visible spectroscopy. A= absorbance.

[1] http://www.cristal.com/ProdDocs/CristalActiV%20PC500%20DataSheet.pdf.

[2] a) Jugan, M. L.; Barillet, S.; Simon-Deckers, A.; Herlin-Boime, N.; Sauvaigo, S.; Douki, T.; Carriere, M., Titanium dioxide nanoparticles exhibit genotoxicity and impair DNA repair activity in A549 cells. Nanotoxicology 2012, 6 (5), 501-13. b) Pignon B; Maskrot H; Leconte Y; Coste S; Reynaud C; Herlin-Boime N; Gervais M; V, G. F.; Pouget T; Tranchant JF, Versatility of laser pyrolysis applied to synthesis of TiO2 nanoparticles, application to UV attenuation. Eur J Inorgan Chem 2008, 208 (6), 883–889; c) Simon-Deckers, A.; Gouget, B.; Mayne-L'hermite, M.; Herlin-Boime, N.; Reynaud, C.; Carriere, M., In vitro investigation of oxide nanoparticle and carbon nanotube toxicity and intracellular accumulation in A549 human pneumocytes. Toxicology 2008, 253 (1-3), 137-46.

[3] a) Arnow, L. E., Colorimetric determination of the components of 3,4-dihydroxyphenylalanine-tyrosine mixtures. J. Biol. Chem 1937, 118, 531; b) Payne, S. M., Detection, isolation, and characterization of siderophores. Methods in enzymology 1994, 235, 329-44.

Section A: Theoretical data

1. Computational Details

Theoretical calculations are made on three types of systems:

- The metal free enterobactin (so called apo), with totally protonated catechol oxygens.
- The metaled enterobactin in which titanium is bound via the six deprotonated oxygen atoms of the three catechol rings (hereafter "catecholate").
- The metaled enterobactin in "salicylate" binding mode, in which three oxygen of the catecholate groups bind the metal while its coordination is completed by the carbonyl oxygens, as presented in figure below. This structure is identified as **ent**-Ti-sal.

Other structures, e.g. partially protonated metal free enterobactins or protonated catecholate structures were addressed but were considered not relevant, especially the latter being less likely to be formed under the experimental conditions.

Metal free enterobactin is a closed-shell, neutral species.

Both metaled structures (catecholate and salicylate binding modes) have a closed shell structure, consistently with the $[Ar]3d^0$ electronic structure of Ti^{4+}. All the calculations have been performed with the Gaussian 09 program [1]. Structures have been optimized with the B3LYP [2] hybrid density functional, completed with Grimme's D3 dispersion correction (keyword: empirical dispersion=GD3BJ) [3], using a double-ζquality basis set constituted as following: the 6-31G* basis [4]set for non-metal atoms and the lanl2dz+ECP one, augmented by a set of polarization functions of exponent 2.462 and a set of d diffuse function of exponent 0.0706 for titanium.

Geometry optimizations, energy calculation and frequency calculation for the simulation of infrared spectra were done in solvent (water), using the IEFPCM implicit model [5].

Infrared transitions are computed by harmonic frequency calculations. A scaling factor of 0.9614 was applied to frequencies to better-fit experimental data [6]. Simulated spectra are obtained by fitting lorentzian functions to peaks provided by the harmonic frequency calculations with a full width at half maximum of 20 cm^{-1}. Vibrational modes (bending, stretching, etc.) have been studied in the range 1000 cm^{-1} to 1800 cm^{-1} and the assignation was made visually by checking vibrational motions. Some modes of interest are coupled and/or delocalized and are attributed to the apparently most involved atoms.

Due to the 3-fold near-symmetric shape of the systems, some modes have virtually the same frequency and are associated to similar vibrations. A typical example concerns the three peaks associated to the C=O stretching modes of the carbonyl groups in the lactone part of the Ti-enterobactin complex with respective frequencies of 1737, 1747 and 1753 cm^{-1} (after scaling) for the salicylate binding mode. This results in a slightly enlarged single peak in the simulated spectrum.

Electronic transitions have been determined using the Time-Dependent Density Functional Theory method [6, 7], on the geometries previously optimized, with the same hybrid functional B3LYP, but with a 6-311++G(d,p) extended basis set, [8] still in solvent. For the three structures, the 50 first singlet- to-singlet transitions have been computed, for the sake of completeness.

2. Optimized structures

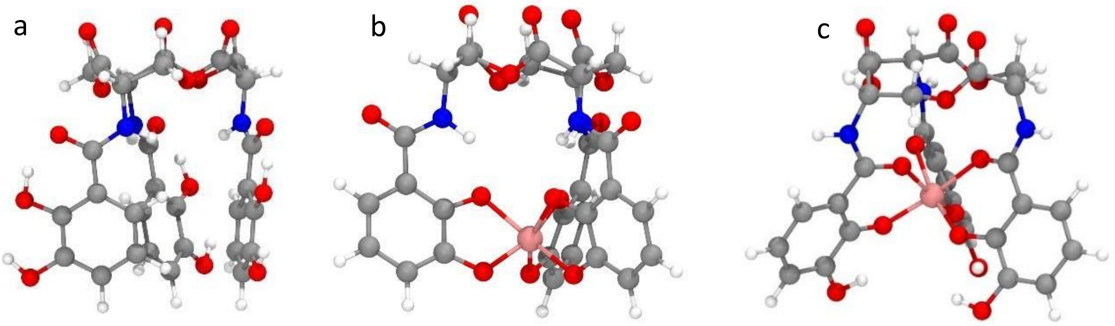

Figure S8. Optimized structures for a: totally protonated apo-enterobactin; b: [Ti(**ent**)]$^{2-}$ complex, in the "catecholate" binding mode and c: [Ti(**ent**H_3)]$^{+}$ complex, in the "salicylate" binding mode. All structures optimized with B3LYP/ [6-31G*; lanl2dz(+,f)+ECP]+D3, IEFPCM implicit water.

a. Simulated UV-Vis spectra

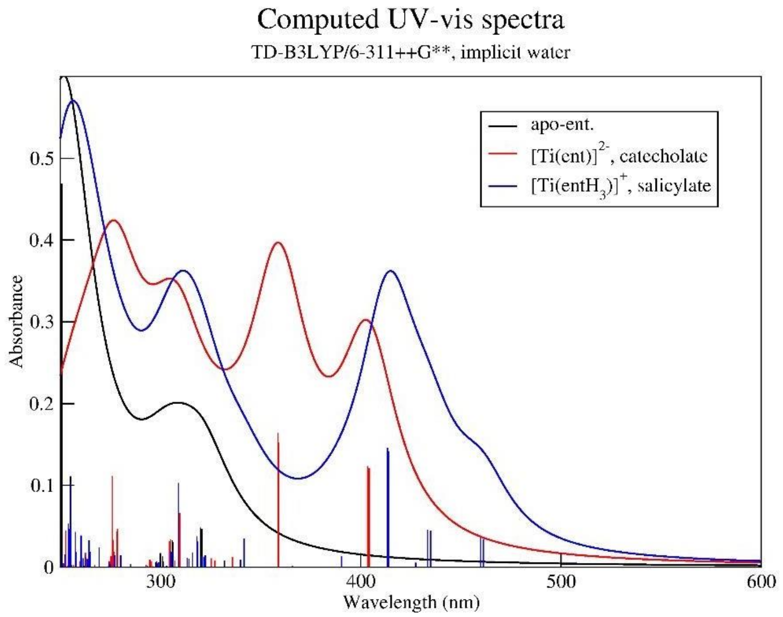

Figure S9. Simulated UV/vis spectra for**:** totally protonated apo-enterobactin (**ent**); (black curve and peaks): [Ti(**ent**)]$^{2-}$ complex, in the "catecholate" binding mode (red) and [Ti(**ent**H_3)]$^{+}$ complex, in the "salicylate" binding mode (blue). Spectra obtained using TD-B3LYP/6-311++G**, implicit water on previously obtained geometries. Curves obtained by fitting lorentzian functions with a half-height width of 30 nm.

b. Simulated IR spectra

a) Computed IR spectrum of the totally protonated enterobactin,

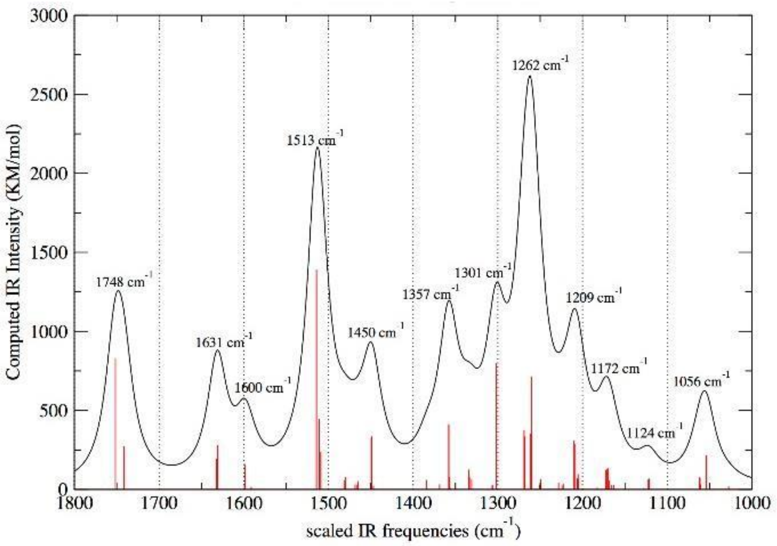

Figure S10. Simulated IR spectrum for the fully protonated enterobactin, Data computed at the B3LYP-D3/double-z + implicit water, all values scaled by 0.9614. Black curve obtained by fitting lorentzian functions with a half-height width of 20 cm^{-1}. Simulated IR spectrum for the totally protonated apo-enterobactin.

Freq of peak (cm^{-1})	Associated lines (cm^{-1})	Experimental (cm^{-1}) (ent in 10v% methanol)	Attribution
**1056**	1026, 1053, 1054	1061, 1074	Internal CC stretch, lactone
**1124**	1120, 1122, 1128	1119, 1139	CN stretch, OH bend. (catechol)
**1172**	1170, 1170, 1172	1178	Internal CC stretch, lactone
**1209**	1205, 1208, 1210	1221, 1236	CH twisting lactone
**1262 (Coupled)**	1260, 1260, 1261<br>1263, 1268, 1269	1259<br>1274	C-O(H) stretching, catechol, CH-bend, lactone
**1301**	1301, 1306, 1306	1294, 1319	C-H bend + N-H bend (amide III)
**1357**	1356, 1357, 1357	1355, 1377	OH bending, catechol
**1450**	1446, 1448, 1449	1441, 1458	C-C stretch, catechol
**1513**	1508, 1511, 1514	1499, 1518	NH bending (amide II)
**1600**	1597, 1597, 1598	1595	C-C stretch, catechol
**1631**	1630, 1630, 1631	1618, 1635	C=O stretch (amide I)
**1748**	1741, 1741, 1752	1738, 1754	C=O stretch, lactone

Table S2. Attribution of peaks for the fully protonated enterobactin with corresponding experimental data. Attribution was made on the basis of visual inspection of related vibrational modes. Data computed at the B3LYP-D3/double-z + implicit water. Original frequencies scaled by 0.9614.

*b) Computed IR spectrum of the [Ti(**ent**H_3)]$^+$ complex, in the "salicylate" binding mode.*

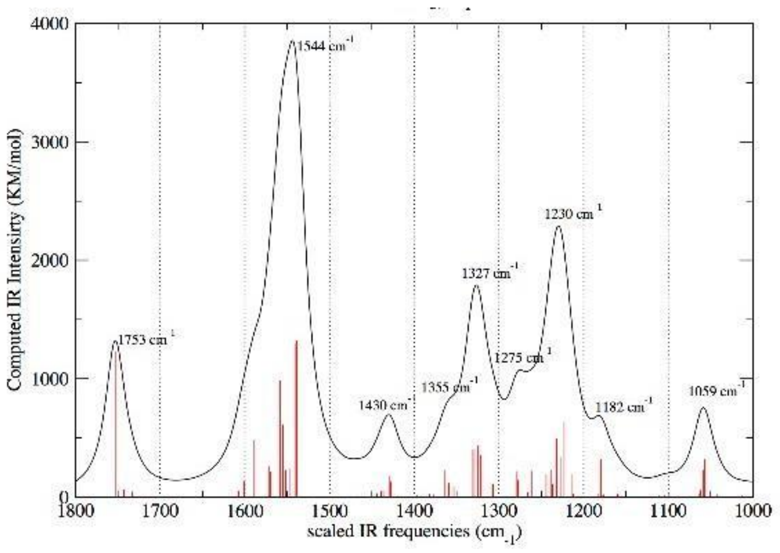

Figure S11. Simulated IR spectrum for the [Ti(**ent**H3)]$^+$ complex, in the "salicylate" binding mode. Data computed at the B3LYP-D3/double-z + implicit water, all values scaled by 0.9614. Black curve obtained by fitting lorentzian functions with a half-height width of 20 cm^{-1}.

**Freq of peak (cm^{-1})**	**Associated lines (cm^{-1})**	**Experimental (cm^{-1}) (Ti-ent complex in PP buffer)**	**Attribution**
**1059**	1041,1056, 1059	1041, 1062, 1070	C-O stretch, CH_2 wagging, lactone
**1182**	1176, 1176, 1179	1139	C-O-H bending catechol
**1230 (coupled)**	1212, 1214, 1222, 1227, 1231, 1231 1236, 1238	1205, 1209, 1222 1224, 1234, 1239 1241,1243	C-O(Ti) stretching, catechol C-H bending, catechol ring
**1275**	1278, 1278, 1279	1271	C-O (H) stretching, catechol ring.
**1327**	1325, 1329, 1331	1323	NH bending (amide III) + CH_2 twist, lactone.
**1355**	1352, 1358, 1363	1357	C-H bending, lactone
**1430**	1427, 1428, 1430	1443, 1453, 1460	C-C stretching + C-O(Ti) stretching, catechol
**1544 (coupled)**	1538, 1541, 1547 1551, 1555, 1558 1570 1588,1600	1519, 1536, 1549 1551, 1554, 1558 1584, 1592, 1614	C=O stretching (amide I) N-H bending (amide II) C-C stretching, catechol ring
**1753**	1737, 1747, 1753	Out of range	C=O, lactone

Table S3. Attribution of peaks for the [Ti(**ent**H_3)]$^+$ complex, in the "salicylate" binding mode, with corresponding experimental data. Attribution was made on the basis of visual inspection of related vibrational modes. Data computed at the B3LYP-D3/double-z + implicit water. Original frequencies scaled by 0.9614

*c) Computed IR spectrum of [Ti(**ent**)]$^{2-}$ complex, in the "catecholate" binding mode*

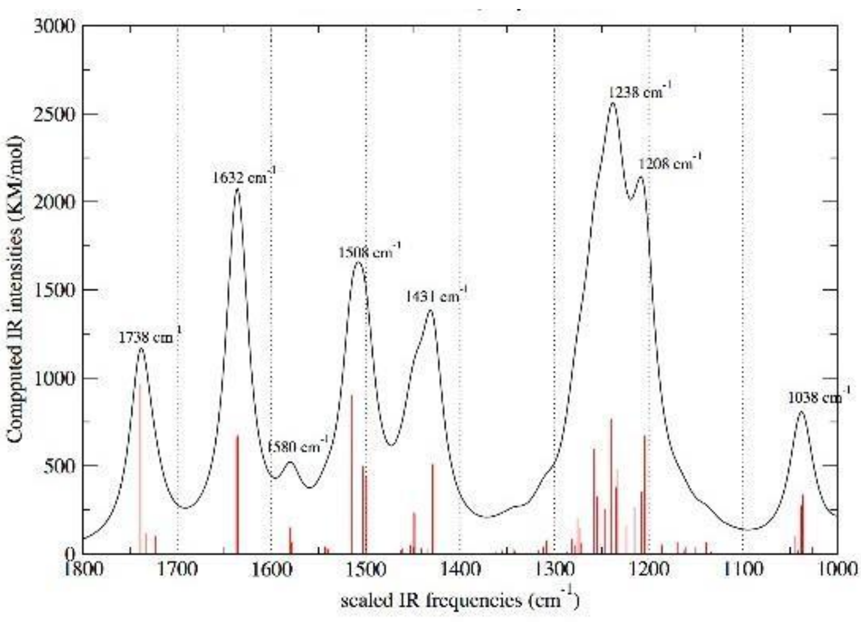

Figure S12. Simulated IR spectrum for the [Ti(**ent**)]$^{2-}$ complex, in the "catecholate" binding mode. Data computed at the B3LYP-D3/double-z + implicit water, all values scaled by 0.9614. Black curve obtained by fitting lorentzian functions with a half-height width of 20 cm^{-1}.

Freq of peak (cm^{-1})	Associated lines (cm^{-1})	Experimental (cm^{-1}) (Supernatant)	Attribution
**1038**	1036, 1038	1033, 1043	C-O stretch, CH_2 wagging, lactone
**1208 (coupled)**	1204, 1204, 1215<br>1207, 1223, 1239	1198, 1219<br>1210, 1228, 1241	C-C stretch, C-O(Ti) stretch, catechol ring<br>CH_2 twist, lactone
**1238**	1233, 1234, 1245	1234, 1244	C-O(Ti) stretch, catechol ring
**1275**[a]	1272,1272,1276	1285	N-H bend (amide III)
**1431**	1429, 1429,1434	1425, 1431, 1438	C-C stretch, catechol ring
**1448**	1448, 1448,1452	1448, 1452, 1459	C-C stretch. + CO(Ti) stretch. Catechole
**1508**	1499, 1502, 1514	1491, 1501, 1508	N-H bend (amide II)
**1580**	1578, 1579, 1580	1570, 1574, 1580	C-C stretch, catechol ring
**1636**	1634, 1635,1638	1634, 1644, 1661	C=O stretch (amide I)
**1738**	1722,1732,1738	Out of range	C=O stretch, lactone

Table S4. Attribution of peaks for the [Ti(**ent**)]$^{2-}$ complex, in the "catecholate" binding mode, with corresponding experimental data. Attribution was made based on visual inspection of related vibrational modes. Data computed at the B3LYP-D3/double-z + implicit water. Original frequencies scaled by 0.9614. (a) : this peak is experimentally observed but is in the foot of the previous one in the simulated spectrum.

References

[1] Frisch, M. J.; Trucks, G. W.; Schlegel, H. B.; Scuseria, G. E.; Robb, M. A.; Cheeseman, J. R.; Scalmani, G.; Barone, V.; Mennucci, B.; Petersson, G. A.; Nakatsuji, H.; Caricato, M.; Li, X.; Hratchian, H. P.; Izmaylov, A. F.; Bloino, J.; Zheng, G.; Sonnenberg, J. L.; Hada, M.; Ehara, M.; Toyota, K.; Fukuda, R.; Hasegawa, J.; Ishida, M.; Nakajima, T.; Honda, Y.; Kitao, O.; Nakai, H.; Vreven, T.; Montgomery, J. A., J.; Peralta, J. E.; Ogliaro, F.; Bearpark, M.; Heyd, J. J.; Brothers, E.; Kudin, K. N.; Staroverov, V. N.; Kobayashi, R.; Normand, J.; Raghavachari, K.; Rendell, A.; Burant, J. C.; Iyengar, S. S.; Tomasi, J.; Cossi, M.; Rega, N.; Millam, J. M.; Klene, M.; Knox, J. E.; Cross, J. B.; Bakken, V.; Adamo, C.; Jaramillo, J.; Gomperts, R.; Stratmann, R. E.; Yazyev, O.; Austin, A. J.; Cammi, R.; Pomelli, C.; Ochterski, J. W.; Martin, R. L.; Morokuma, K.; Zakrzewski, V. G.; Voth, G. A.; Salvador, P.; Dannenberg, J. J.; Dapprich, S.; Daniels, A. D.; Farkas, Ö.; Foresman, J. B.; Ortiz, J. V.; Cioslowski, J.; Fox, D. J., [G09]. In Gaussian 09 Revision D.01, **2009**.

[2] a) Becke, A. D. Density‐functional thermochemistry. III. The role of exact exchange. *J. Chem. Phys.* **1993**, *98*, 5648-5652; b) Lee, C. A.; Yang, W.; Parr, R. G., Development of the Colle-Salvetti correlation-energy formula into a functional of the electron density. *Phys Rev B* **1988**, 37, 785-789; c) Stephens, P. J.; Devlin, F. J.; Chabalowski, C. F.; Frisch, M. J., Ab Initio Calculation of Vibrational Absorption and Circular Dichroism Spectra Using Density Functional Force Fields. *J. Phys. Chem*. **1994**, 98, 11623-11627; d) Vosko, S. H.; Wilk, L.; Nusair, M., Accurate spin-dependent electron liquid correlation energies for local spin density calculations: a critical analysis. *Can. J. Phys.* **1980**, 58, 1200-1211.

[3] Grimme, S.; Ehrlich, S.; Goerigk, L., Effect of the damping function in dispersion corrected density functional theory. *Journal of computational chemistry* **2011**, 32 (7), 1456-65.

[4] a) Hariharan, P. C.; Pople, J. A., The influence of polarization functions on molecular orbital hydrogenation energies. *Theor. Chim. Acta*. **1973**, 28, 213-222; b) Frisch, M. J.; Pople, J. A., Self‐consistent molecular orbital methods 25. Supplementary functions for Gaussian basis sets. *The Journal of chemical physics* **1984**, 80, 3265-3269.

[5] Tomasi, J.; Mennucci, B.; R. Cammi, R., Quantum Mechanical Continuum Solvation Models. *Chem. Rev*. **2005**, 105, 2999-3093.

[6] Scott, A. P.; Radom, L., Harmonic Vibrational Frequencies: An Evaluation of Hartree−Fock, Møller−Plesset, Quadratic Configuration Interaction, Density Functional Theory, and Semiempirical Scale Factors. *J. Phys. Chem.* **1996**, 100, 16502-16513.

[7] a) Bauernschmitt, R.; Ahlrichs, R., Treatment of electronic excitations within the adiabatic approximation of time dependent density functional theory. *Chem. Phys. Lett.* **1996**, 256, 454-464.R; b) Casida, M. E.; Jamorski, C.; Casida, K. C.; Salahub, D. R., Molecular excitation energies to high-lying bound states from time-dependent density-functional response theory: Characterization and correction of the time-dependent local density approximation ionization threshold. *J. Chem. Phys.* **1998**, 108, 4439-4449; c) Stratmann, R. E.; Scuseria, G. E.; Frisch, M. J., An efficient implementation of time-dependent density-functional theory for the calculation of excitation energies of large molecules. *J. Chem. Phys.* **1998**, 109, 8218-8224.

[8] a) 1. McLean, A. D.; Chandler, G. S., Contracted Gaussian basis sets for molecular calculations. I. Second row atoms, Z=11–18. *J. Chem. Phys.* **1980**, 72, 5639-5648. b) Raghavachari, K.; Binkley, J. S.; Seeger, R.; Pople, J. A., Self-consistent molecular orbital methods. XX. A basis set for correlated wave functions. *J. Chem. Phys.* **1980**, 72, 650-654.

Section B: XPS analysis of the TiO_2 NPs interaction with Enterobactin

The elemental composition and chemical states of A12 and P25 samples before and after incubation with enterobactin for 24 h were evaluated by XPS that allowed us to analyze the surface of titanium powders. The XPS survey spectra of the samples revealed prominent peaks of carbon (C1s), oxygen (O1s) and titanium (Ti2p) (Figure S13). After incubation with **ent** for 24 h, the presence of nitrogen (N1s) and phosphate (P2p) peaks (Figure S13b,d) confirms the TiO_2 nanoparticles surface modification. In adition, nitrogen (N1s) and chlorine (Cl2p) detected in the XPS survey spectra of P25 sample before incubation with **ent** (Figure S13a) could be due to contamination during the synthesis process.

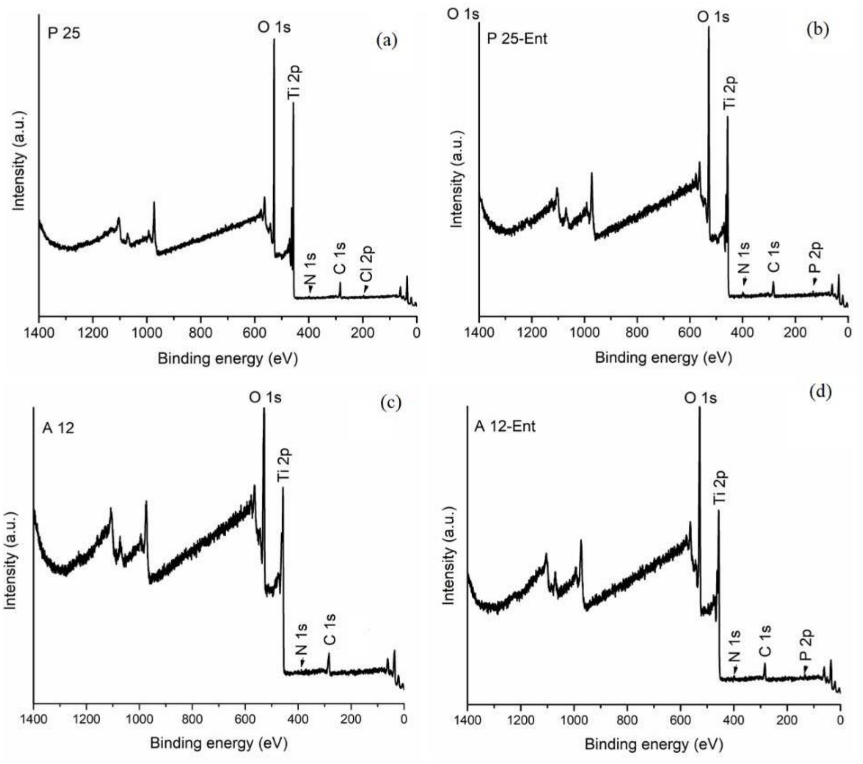

Figure S13. XPS survey spectra of the P 25 (a, b) and A 12 (c, d) samples analyzed before (a, c) and after (b, d) incubation with enterobactin for 24h.

The high resolution spectra of the C1s peaks of A12 and P25 samples before and after incubation with **ent** for 24 h were presented in Figure S14. The binding energy scale was calibrated to adventitious C1s at 284.8 eV. The C1s XPS high resolution spectra have been deconvoluted into three to five components. Before incubation with **ent** of both A12 and P25 samples, the biggest peaks observed in C1s high resolution spectra were located at BE of 284.8 3V and were assigned to the C-C/C-H bonds [1]. The peaks located at BE at 286. 32 eV in C1s high resolution spectra are attributed to (C–O) [2]. The peak observed at BE of 288.94 eV in C1s high resolution spectra of P 25 sample before incubation (Figure S14a) could be allocated to NC=O bonds [3]. The peak located at BE at 283.38 eV in the C1s region of A12 sample before incubation (Figure S14c) was assigned to C-C bonds [4] while the peaks located at BEs of 287.67 eV and 289.48 eV were assigned to C=O/O–C–O and O-C=O [2]. After incubation with **ent** of both A12 and P25 samples, the three most important components were located at BEs of 283.48 eV, 284.8 eV, and 286.34 eV and were assigned to C-

C bonds [3], C-C/C-H bonds [1], and C–O bonds [2]. The component of high resolution spectra of P25 sample after incubation with **ent** located at BE of 287.78 eV was assigned to bonds O-C= O bonds [2], while the component of high resolution spectra of A12 sample after incubation with **ent** located at BE of 288.32 eV was assigned C= O bonds [5].

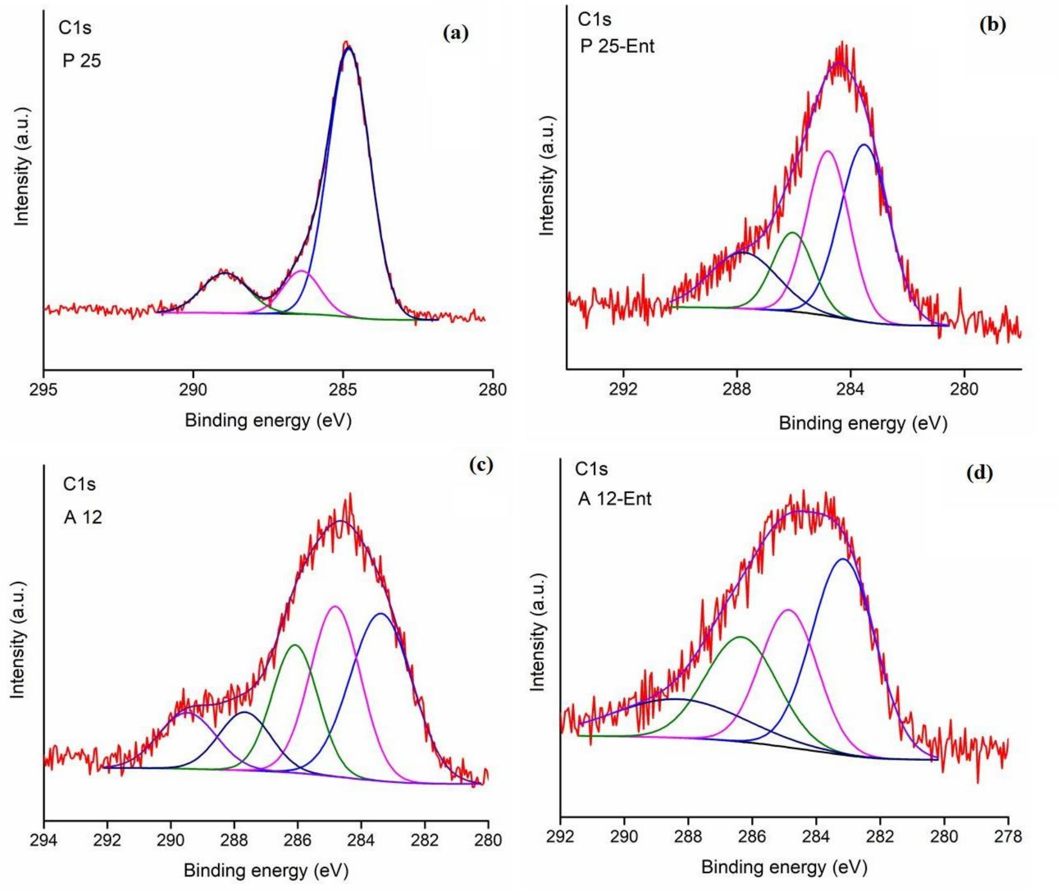

Figure S14. High-resolution XPS spectra of the C1s peaks of the P25 (a, b) and A12 (c, d) samples before (a, c) and after (b,d) incubation with **ent** for 24h.

Figure S15 corresponds to the high resolution spectra of the O1s peaks of the P25 and A12 samples before and after incubation with **ent** for 24 h. The high resolution spectrum O1s have been deconvoluted with three and four components for the P25 and A12 samples, respectively. The components of O1s located at binding energies (BEs) of 528.5 eV (Figure S15b), 528.22 eV (Figure S15c) and 528.31 eV (Figure S15d) could be attributed to the oxygen in the well-known surface reconstructions on TiO_2 nanoparticles [6-7] that gives rise to O1s features consistent with nucleophilic oxygen at 528.1–528.5 eV [8]. This peak was not observed in the O1s spectrum of the P25 sample before incubation (Figure S15a). For A12, this species of oxygen was represented by a peak localized around 528 eV and increased after incubation with **ent** (Figure S15d). The deconvoluted O1s high resolution spectra revealed additional peaks at BEs of 529.98 eV (Figure S15a), 529.75 eV (Figure S15b), 529.51 eV (Figure S15c) and 529.64 eV (Figure S15d), that can be assigned to the Ti-O bonds **[9]** and to crystal lattice oxygen in O-Ti-O linkage [10]. According with previous studies [11], the peaks observed at 529.75 eV (Figure S15b) and 529.64 eV (Figure S15d) could also be assigned to the presence of P- O-Ti linkage in the A12 and P 25 samples after incubation with **ent**. The observed peaks at BEs around 530 eV can be assigned to -OH groups as a result of chemisorbed H_2O [12] (530.61 eV in Figure 3c, 530.93 eV in

Figure S15a and 530.92 eV in Figure S15b). The peaks located at BEs of 531.64 eV for A12 sample before incubation (Figure S15c) can be attributed to C-O bonds [13-14]. The peaks located at BEs of 531.02 eV for A12 sample after incubation (Figure S15d) can be attributed Ti-OH bonds [15-17]. O1s spectra of the A12 sample after incubation (Figure S15d) revealed also a small contribution located at around 532.32 eV that can be assigned to the chemisorbed oxygen species [12] orto P-O-P bonds [18]. On the other hand, previous studies [19, 20] have shown that high-binding-energy oxygen sub-peaks at BEs at 532.0 eV and 530.32 eV could occur from a lack of electrons in the non-stoichiometric oxygen atom, which corresponds to the oxygen in the Ti–O–N or Ti–N–O bonds, that being in good agreement with our results. A tiny contribution located at BE of 532.0 eV was identified for P25 sample before incubation but not for A12 sample before incubation and P25 sample after incubation.

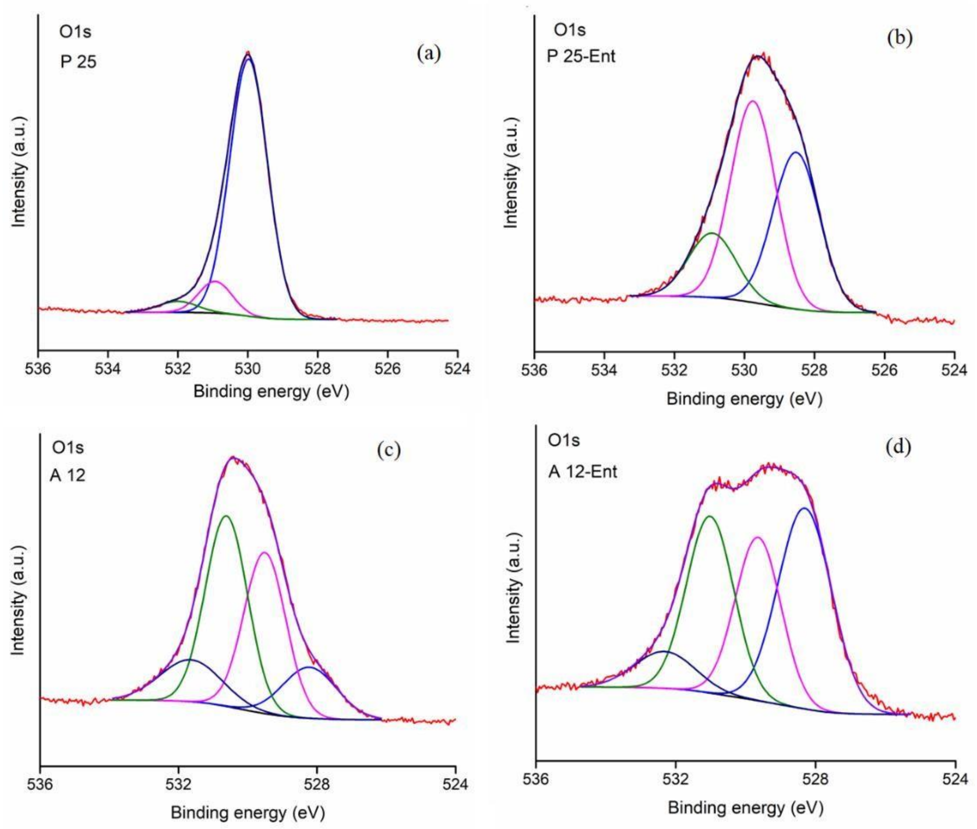

Figure S15. High-resolution XPS spectra of the O1s peaks of the P25 (a, b) and A12 (c,d) samples before (a, c) and after (b, d) incubation with **ent** for 24h.

The high-resolution spectra of Ti2p peaks of the P25 and A12 samples before and after incubation with **ent** were assessed in Figure S16.

The high-resolution spectra of Ti2p peak of the P25 sample before incubation (Figure S16a) revealed a single component after deconvolution at BEs of 458.74 eV and 464.47 eV with a peak separation of 5.73 eV. The peaks correspond to the Ti $2p_{3/2}$ and Ti $2p_{1/2}$ and indicate Ti^{4+} species are present in the sample in agreement with previous studies [21, 22].

After incubation with **ent** for 24h (Figure S16b) the peaks were wider and shifted to smaller BEs which led to its deconvolution in two components. The first component was located at BEs of 458.55 eV and 464.25 eV suitable to the Ti $2p_{3/2}$ and Ti $2p_{1/2}$ levels of Ti^{4+} in TiO_2 [21] with a peaks separation value of 5.7 eV in agreement with precedent literature [22]. The second component located at lower binding energies with

values of 457.20 eV and 462,898 eV highlights the presence of Ti^{3+} species [4]. This behavior is similar to the case where the surface of sample P25 was changed [21].
For A12 samples before incubation, Figure S16c shows peaks at 458.73 eV and 457.09 eV assigned to the $Ti2p_{3/2}$, and peaks at 464.50 eV and 462.80 eV assigned to $Ti2p_{1/2}$, respectively, related to the level of Ti^{4+} and Ti^{3+} [24-25]. For A12 sample after incubation (Figure S16d) the spectrum two components, which correspond to the Ti $2p_{3/2}$ (458.32 eV and 459.65 eV) and Ti $2p_{1/2}$ (464.27 eV and 465.57 eV) levels showing that the oxidation state of titanium is Ti^{4+} [26-27]. The peak separation of 5.92 eV between the Ti $2p_{1/2}$ and Ti $2p_{3/2}$ signals was in accordance with the literature [28]. On the other hand, the origin of the last component locate at BEs of 457.01 eV and 462.79 eV was associated to $Ti2p_{3/2}$ and $Ti2p_{1/2}$ levels of Ti^{3+} in TiO_2 [24, 27].
From the XPS spectra shown in Figure S16 it can be seen that the proportion of Ti^{3+} increases in both samples after being incubated with **ent** for 24h, especially in A12 sample. In recent studies, Y.J. Hwang et al. [29] demonstrated that the proportion of Ti^{3+} increases in the presence of oxygen vacancies, which indicates that the intensity of the Ti^{3+} peak is related to surface defects.

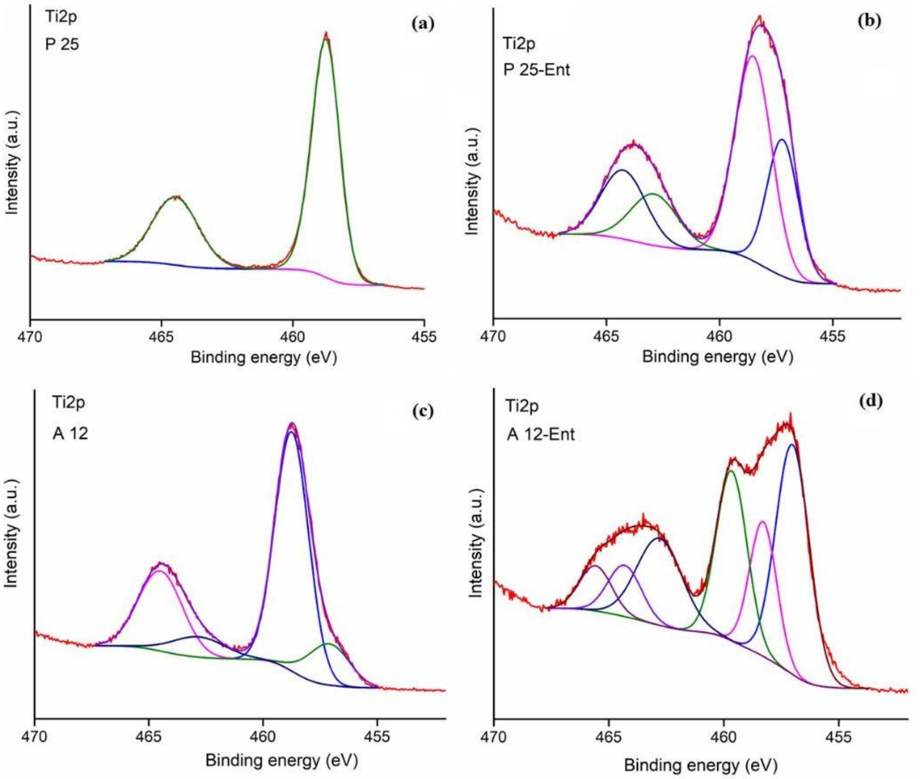

Figure S16. High-resolution XPS spectra of the Ti2p peaks of the P25 (a, b) and A12 (c, d) samples before (a, c) and after (b, d) incubation with **ent** for 24 h

It should be noted that in the XPS survey spectra (Figure S13) of the P25 and A12 samples collected before and after incubation with **ent** for 24 h a peak of N1s was identified. The corresponding N1s high resolution spectra were presented in Figure S17. Consistent with previous studies [30-31], N may be located in interstitial and/or substitution sites. According to the studies of R.G. Palgrave et al. [31], the peaks in the range 395.5-397 eV can be attributed to substitutive nitrogen (**Nsub**). F. Zhou et al. [32] and Y. Cong et al. [33] showed that peaks in the range of 398-400 eV could be attributed to interstitial nitrogen (**Nint**). The N1s high resolution spectrum of P25 and A12 before incubation presented a single component centered at 400.08 eV (Figure S17a) and 399.61 eV (Figure S17c) that could be attributed to the interstitial nitrogen (**Nint**)[32-33]. On the other hand, according to previous studies reported by A. Gao et al. [34] we could say that

the presence of N in P25 and A12 samples before incubation (Figure S17a, c) could be attributed to surface contamination during the process of obtaining of TiO_2 powders. In the case of the P25 sample after incubation, two peaks were highlighted (Figure S17b) centered at 398.62 eV and 400.23 eV that could be attributed to the interstitial nitrogen (**Nint**) [32-33]. For A12 sample after incubation , Figure S17d showed three peaks centered at 397.96 eV, 399.61 eV and 401.23 eV. The first two peaks could be attributed to substitutive nitrogen (**Nsub**) and interstitial nitrogen (**Nint**). According to previous studies [35-36], the peak centered at BE of 401.23 eV could be due to N1s binding energy in the environment of O-Ti -N.

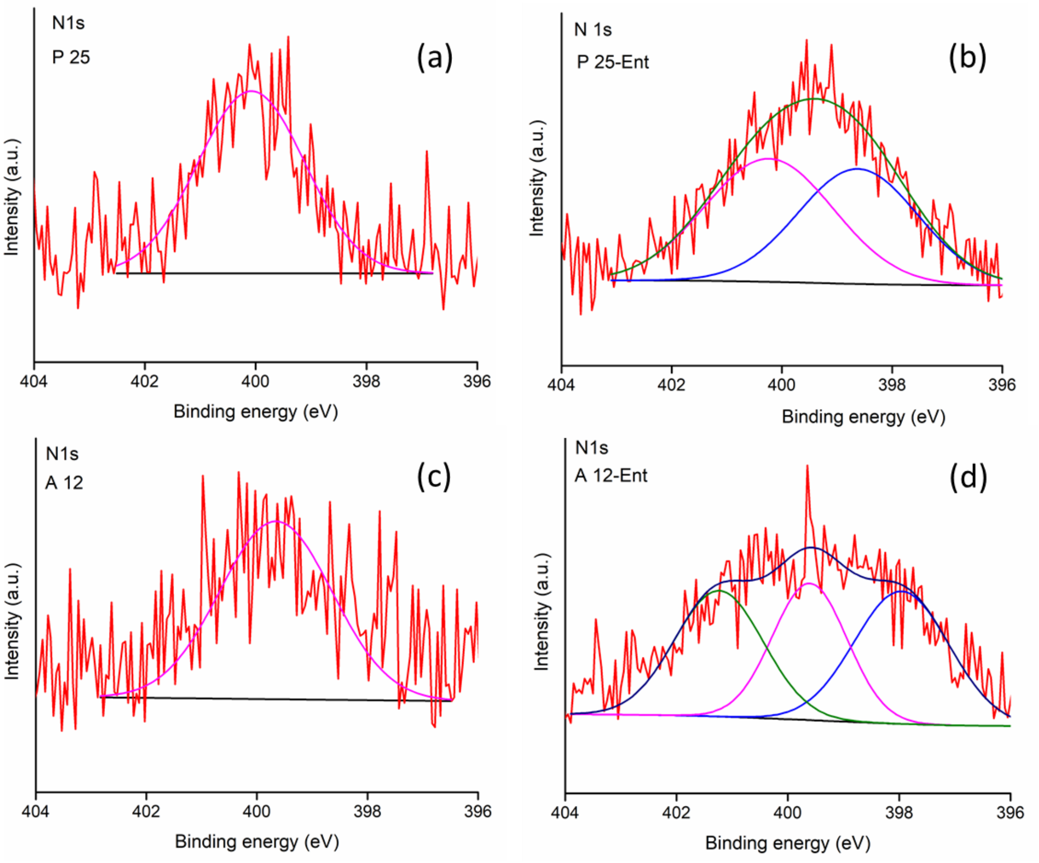

Figure S17. High-resolution XPS spectra of the N1s peaks of the P25 (a, b) and A12 (c,d) samples before (a, c) and after (b, d) incubation with **ent** for 24h.

The XPS survey spectra of the samples shows some Chlorine corresponding signal in the P25 sample. According to previous studies the presence of traces of Cl appears to be connected to the rutile phase, presents in P25 but not in A12 [37].

The high resolution spectra of the P2p peaks of P25 and A12 samples after incubation with **ent** were shown in Figures S18. It shows that some phosphate is bounded to the surface of TiO_2 particles. The peaks located at BEs of 132.2 eV in XPS spectra of the P2p of P25 sample can be associated to the $P2p_{1/2}$ orbital of phosphorus (Figure S18b). The P2p orbital of the A12 sample shift to 132.9 eV (Figure S18a). According to last studies [38], the peak located at BE of 132.2 eV can be attributed to the PO_4^{3-} group (the P^{5+} valence state). The BE at 133.85 eV in P25 incubated with **ent** was assigned to the $P2p_{3/2}$ component that indicate the

presence of phosphate with only one hydroxyl unit (Figure S18b). In comparison with the position of the P2p component observed in the A12 sample after incubation (Figure S18a) a shift to 134.59 eV was observed. In agreement with the previous studies [39], the BE at 134.5 eV suggests that the P2p identified could be due to the P in a pentavalent oxidation state indicated by P^{5+}. This studies have shown a slight increase in binding energy for samples incubated with **ent** for 24 hours.

On the other hand, the peak espected around 128.6 eV characteristic of the binding energy for Ti-P [40] was not detected in the samples after incubation with **ent** for 24h (Figure S18). Since P has an ionic radius of 0.35 Å it could replace the Ti atoms (knowing that the ionic radius of Ti is 0.67 Å). Phosphate could be then present in the form of Ti-O-P (as been observed in the high resolution spectra of O1s by the presence of peaks located at BEs of 529.75 eV (Figure S15b) and 529.64 eV (Figure S15d)). Moreover, previous studies [41] have shown that surface phosphorus species can contribute to the improvement properties of doped TiO_2. From the analysis of the high resolution spectra of P2p of P25 and A12 samples after incubation with **ent** for 24h that shows no Ti-P it confirms that the peaks that appear at the binding energies of 457. 2 eV (Figure S16b) and 457.09 eV (Figure S16d) are a indicative for Ti^{3+} that is formed after reduction of Ti^{4+}. The presence of these peaks indicates that part of Ti has been reduced highlighting the presence of surface oxygen vacancies [42].

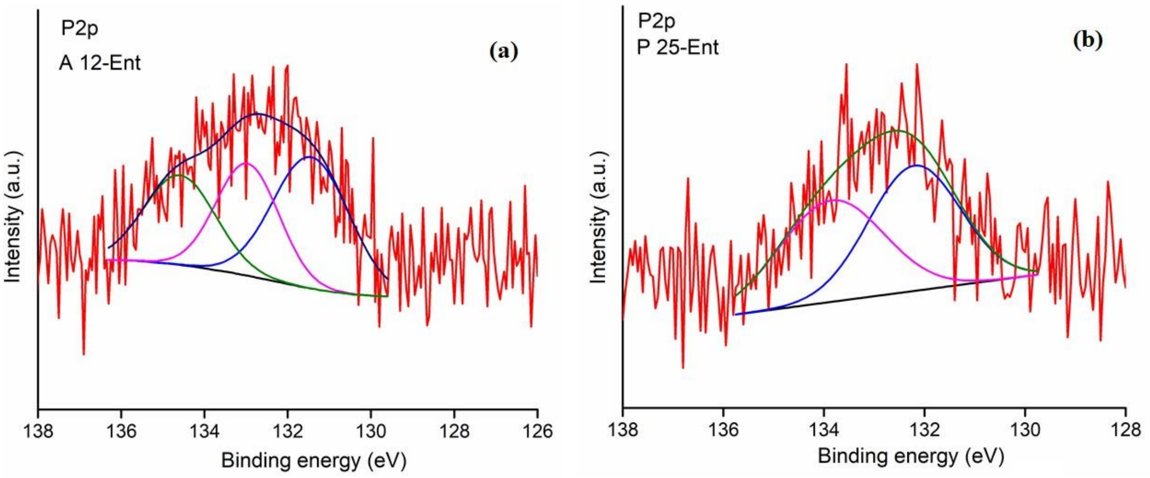

Figure S18. High-resolution XPS spectra of the P2p peaks of the A12 (a) P25 (b) and samples after incubation with **ent** for 24h.

In summary, from the analysis of XPS, it is clear that changes are observed after the interaction with enterobactin. This one is thus bound to the surface. Furthermore, Ti^{4+} but also a little Ti^{3+} signatures are present in the spectra. Ti^{3+} increased in intensity after incubation of both P25 and A12 with **ent** for 24h related to surface defects due to the presence of oxygen vacancies. For A12 incubated with ent, Ti-OH signature has been observed in O1s spectrum. Furthermore, nitrogen and phosphorus elements (P^{5+} oxidation state in $PO_4^{2-/3-}$) are present in the A12 and P25 sample before and after incubation with **ent** for 24h, and some chlorine in P25, all probably due to surface contamination during the process of obtaining of TiO_2 powders.

References:

[1] Barr, T. L. and Seal, S. Nature of the use of adventitious carbon as a binding energy standard *Journal of Vacuum Science & Technology A* **1995,** 13, 1239. doi.org/10.1116/1.579868J.

[2] Miller, D. J.; Biesinger, M. C.; McIntyre, N. S. Interactions of CO_2 and CO at fractional atmosphere pressures with iron and iron oxide surfaces: one possible mechanism for surface contamination? *Surf. Interface Anal*. **2002**, 33, 299–305. doi:10.1002/sia.1188.

[3] Sobaszek M**.**, Siuzdak K., Sawczak M., Ryl J., Bogdanowicz R., Fabrication and characterization of composite TiO2 nanotubes/ boron-doped diamond electrodes towards enhanced supercapacitors, *Thin Solid Films*, 2016, 601, 35-40**, doi.org/10.1016/j.tsf.2015.09.073**.

[4] Suárez L., Pulgarin C., Roussel C., Kiwi J., Preparation, kinetics, mechanism and properties of semi-transparentphotocatalytic stable films active in dye degradation, *Applied Catalysis A: General*, **2016**, 516, 70–80, doi.org/10.1016/j.apcata.2016.01.041

[5] Al-Gaashania, R.; Najjar, A.; Zakaria, Y.; Mansour, S.; Atieh M.A., XPS and structural studies of high quality graphene oxide and reduced graphene oxide prepared by different chemical oxidation methods, *Ceramics International*, **2019**, 45(11), 14439-1444, DOI: 10.1016/j.ceramint.2019.04.165.

[6] Carlisle, C.; Fujimoto, T.; Sim, W.; King, D. Atomic imaging of the transition between oxygen chemisorption and oxide ®lm growth on Ag{1 1 1}. *Surf. Sci.* **2000**, 470 , 15 –31, DOI:10.1016/S0039-6028(00)00831-1.

[7] Bare, S. R.; Griffiths, K.; Lennard, W. N.; Tang, H. T., Generation of atomic oxygen on Ag(111) and Ag(110) using NO_2: a TPD, LEED, HREELS, XPS and NRA study. *Surf. Sci.* **1995** , 342, 185–198. doi.org/10.1016/0039-6028(95)00670-2.

[8] Jones, T. E. Tulio C. R. Rocha, Axel Knop-Gericke, Catherine Stampfl, Robert Schlögl, and Simone Piccinin, Insights into the Electronic Structure of the Oxygen Species Active in Alkene Epoxidation on Silver, *ACS Catalysis* **2015**, 5, 5846−5850, doi: 10.1021/acscatal.5b01543.

[9] Yu, J.C.; Yu, J.; Ho, W.; Jiang, Z.; Zhang, L., Effects of F- Doping on the Photocatalytic Activity and Microstructures of Nanocrystalline TiO_2 Powders. *Chem. Mater*. **2002**, 14, 3808-3816, doi: 10.1021/cm020027c.

[10] Assayehegn, E.; Solaiappan, A. ;Chebude, Y.; Alemayehu, E., Fabrication of tunable anatase/rutile heterojunction N/TiO_2 nanophotocatalyst for enhanced visible light degradation activity, *Applied Surface Science* **2020,** 515, 145966. Doi 10.1021/cm020027c.

[11] Joseph Antony Raj, K.; Shanmugam, R.; Mahalakshmi, R.; Viswanathan, B., XPS and IR spectral studies on the structure of phosphate and sulphate modified titania – A combined DFT and experimental study, *Indian Journal of Chemistry* 2010, 49A, 9-17.

[12] Zhang, J.; Chen, C.; Lu, H.; Leng, D., Li, G.; Liu, Y.; Liang, Q.; Gao, J.; Wang, C.; Zhu, B., Construction of anatase@rutile core@shell TiO_2 nanosheets with controllable shell layer thicknesses for enhanced ethanol sensing, *Sensors and Actuators: B. Chemical*, **2020**, 325, 128815.

[13] Moulder, J.F.; Stickle,W.F.; Sobol, P.E.; Bomben, K.D. Handbook of X-Ray Photoelectron Spectroscopy; Perkin-Elmer Corporation: Eden Prairie, MN, USA, **1992**.

[14] Wang, P.H.; Yap, P.S.; Lim, T.T. C–N–S tridoped TiO_2 for photocatalytic degradation of tetracycline under visible-light irradiation. *Appl. Catal. A* **2011**, 399, 252–261, doi: 10.1016/j.apcata.2011.04.008.

[15] Sanjines, R.; Tang, H.; Berger, F.; Gozzo, F.; Margaritonto, G.; Levy, F. J., Electronic structure of anatase TiO2 oxide. *Journal of Applied Appl. Phys*. **1994**, 75 (6), 2945, doi: 10.1063/1.356190.

[16] Riakar, G. N.; Gregory, J. C.; Ong, J. L.; Lucas, L. C.; Lemons, J. E.; Kawahara, D.; Nakamura, M. *J. Vac. Sci. Technol.* **1995**, 13 (5), 2633.

[17] Yu, Y.P.; Xing, X.J.; Xu, L.M.; Wu, S.X.; Li, S.W., N-derived signals in the X-ray photoelectron spectra of N-doped anatase TiO_2. *J. Appl. Phys.* **2009**, 105, 123535, doi.org/10.1063/1.3143882.

[18] Thogersen, A.; Syre, M.; Retterstol Olaisen, B.; Diplas, S., Studies of the oxidation states of phosphorus gettered silicon substrates using X-ray photoelectron spectroscopy and transmission electron microscopy, *Journal of Applied Physics* **2013**, 113. /doi/10.1063/1.4775818.

[19] Zajac, A.T.; Radecka, M.; Zakrzewska, K.; Brudnik, A.; Kusior, E.; Bourgeois, S.; Marco de Lucas, M.C.; Imhoff, L. Structural and electrical properties of magnetron sputtered Ti(ON) thin films: The case of TiN doped in situ with oxygen. *J. Power Sources* **2009**, 194, 93–103, doi: 10.1016/j.jpowsour.2008.12.112.

[20] Lahoz, R.; Espinós, J.P.; Fuente, G.F.D.; Elipe, A.R.G. "in situ" XPS studies of laser induced surface cleaning and nitridation of Ti. *Surf. Coat. Tech.* **2008**, 202, 1486–1492, doi: 10.3390/coatings10010047.

[21] Erdem, B.; Hunsicker, R.A.; Simmons, G.W.; Sudol, E.D.; Dimonie, V.L. and El-Aasser M.S., XPS and FTIR Surface Characterization of TiO_2 Particles Used in Polymer Encapsulation, *Langmuir* **2001**, 17, 2664-2669. Doi 10.1021/la0015213.

[22] http://www.xpsfitting.com/2008/09/titanium.html

[23] Tshabalala, Z.P.; Shingange, K.; Dhonge, B.P.; Ntwaeaborwa, O.M.; Mhlongo, G.H.; Motaung, D.E. Fabrication of ultra-high sensitive and selective CH4 room temperature gas sensing of TiO_2 nanorods: Detailed study on the annealing temperature, *Sens. Actuators B* **2017**, 238,402–419, doi.org/10.1016/j.snb.2016. 07.023

[24] NIST X-ray Photoelectron Spectroscopy Database. Available online: https://srdata.nist.gov/xps/ (accessed on 26 April 2019).

[25] García Guillén, G.; Shaji, S.; Mendivil, I.; Avellaneda, D.; Castillo, G.; Roy, T.; Garcia Gutierrez, D.; Krishnan, B., Effects of ablation energy and post-irradiation on the structure and properties of titanium dioxide nanomaterials, *Appl. Surf. Sci*. **2017**, 405, 183-194. doi.org/10.1016/j.apsusc. 2017.01.282

[26] Wang, X.; Hu, Z.; Chen, Y.; Zhao, G.; Liu, Y.; Wen, Z., A novel approach towards high performance composite photocatalyst of TiO_2 deposited on activated carbon. *Appl Surf Sci* 2009, 255:3953–3958

[27] Butler, E.B.; Chen, C.C.; Hung, Y.T.; Al Ahmand, M.S. Effect of Fe-doped TiO2 photocatalysts on the degradation of acid orange 7. *Integrated Ferroelectrics* **2016**, 168, 1-9. doi.org/10.1080/ 10584587.2016.1157779.

[28] McCafferty, E.; Wightman, J. P. Determination of the concentration of surface hydroxyl groups on metal oxide films by a quantitative XPS method. *Surf. Interface Anal*. **1998**, 26, 549-564.

[29] Hwang, Y.J.; Yang, S.; Lee, H., Surface analysis of N-doped TiO_2 nanorods and their enhanced photocatalytic oxidation activity. *Appl. Catal. B Environ.* **2017**, 204, 209-215, doi: 10.1016/j.apcatb.2016.11.038.

[30] Liu, S.; Cai, Z.; Zhou, J. Pan, A., Liang, S., Nitrogen-doped TiO_2 nanospheres for advanced sodium-ion battery and sodium-ion capacitor applications, *J Mater. Chem. A* **2016**, 4, 18278–18283.

[31] Palgrave, R.G.; Payne, D.J.; Egdell, R.G., Nitrogen diffusion in doped TiO_2 (110) single crystals: a combined XPS and SIMS study, *J. Mater. Chem.* **2009,** 19, 8418–8425, doi: 10.1039/C6TA08472A.

[32] Zhou, F.; Song, H.; Wang, H.; Komarneni, S.; Yan, C., N-doped TiO_2/sepiolite nanocomposites with enhanced visible-light catalysis: role of N precursors, *Appl. Clay Sci*. **2018**, 166, 9–17, doi: 10.1016/j.clay.2018.08.025.

[33] Cong, Y.; Zhang, J.; Chen, F.; Anpo, M., Synthesis and characterization of nitrogendoped TiO_2 nanophotocatalyst with high visible light activity, *J. Phys. Chem. C.* **2007**, 111, 6976–6982, 10.1021/jp0685030.

[34] Gao, A.; Hang, R.; Huang, Zhao, X. L. Zhang, X.; Wang, L.; Tang, B.; Ma, S.; Chu, P.K., The effects of titania nanotubes with embedded silver oxide nanoparticles on bacteria and osteoblasts, *Biomaterials* **2014**, 35, 4223-4235, 10.1016/j.biomaterials.2014.01.058.

[35] Periyat, P.; McCormack, D.E.; Hinder, S.J.; Pillai, S.C., One-Pot Synthesis of Anionic (Nitrogen) and Cationic (Sulfur) Codoped High-Temperature Stable, Visible Light Active, Anatase Photocatalysts, *J. Phys. Chem. C.* **2009,** 113, 3246–3253. doi:10.1021/jp808444y.

[36] Peng, F.; Cai, L.; Yu, H.; Wang, H.; Yang, J., Synthesis and characterization of substitutional and interstitial nitrogen-doped titanium dioxides with visible light photocatalytic activity, *J. Solid State Chem*. **2008,** 181,130–136. doi:10.1016/j.jssc.2007.11.012.

[37] Gálvez-López, M.F.; Muñoz-Batista, M.J.; Alvarado-Beltrán, C.G.; Almaral-Sánchez, J.L.; Bachiller-Baeza, B.; Kubacka, A.; Fernández-García, M., Sn modification of TiO_2 anatase and rutile type phases: 2-Propanol photooxidation under UV and visible light, *Applied Catalysis B: Environmental*, **2018**, 228, 130–141, doi: 10.1016/j.apcatb.2018.01.075.

[38] Ramana, C.V.; Ait-Salah, A.; Utsunomiya, S.; Morhange, J.-F.; Mauger, A.; Gendron, F.; Julien, C.M., Spectroscopic and chemical imaging analysis of lithium iron triphosphate, *J. Phys. Chem. C* **2007,** 111, 1049–1054 (), http://dx.doi.org/10.1021/ jp065072c.

[39] Nakamura, R.; Tanaka, T.; Nakato, Y., Mechanism for Visible Light Responses in Anodic Photocurrents at N-Doped TiO_2 Film Electrodes, *J. Phys. Chem. B.* **2004,** 108, 10617–10620. doi:10.1021/jp048112q.

[40] Baunack, S.; Oswald, S.; Scharnweber, D., Depth distribution and bonding states of phosphorus implanted in titanium investigated by AES, XPS and SIMS, *Surf. Interface Anal.* **1998,** 26, 471–479. doi:10.1002/(SICI)1096-9918(19980515)26:63.0.CO;2-S.

[41] Zheng, R.; Guo, Y.; Jin, C.; Xie, J.; Zhu, Y.; Xie, Y., Novel thermally stable phosphorus-doped TiO_2 photocatalyst synthesized by hydrolysis of TiCl4, *J. Mol. Catal. A Chem*. **2010**, 319,46–51. doi:10.1016/j.molcata.2009.11.

[42] Nolan, N.T.; Seery, M.K.; Hinder, S.J.; Healy, L.F.; Pillai, S.C., A systematic study of the effect of silver on the chelation of formic acid to a titanium precursor and the resulting effect on the anatase to rutile transformation of TiO_2, *J. Phys. Chem. C.* **2010,** 114, 13026–13034. doi:10.1021/jp1016054.
